# Characterizing haploinsufficiency of *SHELL* gene to improve fruit form prediction in introgressive hybrids of oil palm

**DOI:** 10.1038/s41598-017-03225-7

**Published:** 2017-06-08

**Authors:** Chee-Keng Teh, Siti Dalila Muaz, Praveena Tangaya, Po-Yee Fong, Ai-Ling Ong, Sean Mayes, Fook-Tim Chew, Harikrishna Kulaveerasingam, David Appleton

**Affiliations:** 1 Biotechnology & Breeding Department, Sime Darby Plantation R&D Centre, Selangor, Malaysia; 2 School of Biosciences, University of Nottingham, Nottingham, UK; 3 Department of Biological Sciences, National University of Singapore, Singapore, Singapore

## Abstract

The fundamental trait in selective breeding of oil palm (*Eleais guineensis* Jacq.) is the shell thickness surrounding the kernel. The monogenic shell thickness is inversely correlated to mesocarp thickness, where the crude palm oil accumulates. Commercial thin-shelled *tenera* derived from thick-shelled *dura* × shell-less *pisifera* generally contain 30% higher oil per bunch. Two mutations, *sh* ^*MPOB*^ (M1) and *sh* ^*AVROS*^ (M2) in the *SHELL* gene – a type II MADS-box transcription factor mainly present in AVROS and Nigerian origins, were reported to be responsible for different fruit forms. In this study, we have tested 1,339 samples maintained in Sime Darby Plantation using both mutations. Five genotype-phenotype discrepancies and eight controls were then re-tested with all five reported mutations (*sh* ^*AVROS*^, *sh* ^*MPOB*^, *sh* ^*MPOB2*^, *sh* ^*MPOB3*^ and *sh* ^*MPOB4*^) within the same gene. The integration of genotypic data, pedigree records and shell formation model further explained the haploinsufficiency effect on the *SHELL* gene with different number of functional copies. Some rare mutations were also identified, suggesting a need to further confirm the existence of cis-compound mutations in the gene. With this, the prediction accuracy of fruit forms can be further improved, especially in introgressive hybrids of oil palm. Understanding causative variant segregation is extremely important, even for monogenic traits such as shell thickness in oil palm.

## Introduction

*Elaeis*, the only genus under the Arecaceae family, produces edible oil. *Elaeis guineensis* Jacq. of West African origin is the predominantly used species, planted due to oil yield superiority, whereas the planting of another related species, *E*. *oleifera* (HBK) Cortes is usually restricted to Latin America, where the species originated from. In oil palm, three naturally occurring fruit forms (*dura*, *tenera* and *pisifera*) can be recognized based on their shell thickness, which is controlled by a single gene with two co-dominant alleles^1^. The thinner shell usually confers additional mesocarp to the fruit, allowing more oil to accumulate. Hence, planters in Southeast Asia in the 1960s switched from planting Deli *dura* (thick-shelled) fruit to *tenera* (thin-shelled) fruit by making hybrids between Deli *dura* and AVROS *pisifera* (shell-less, typically female sterile), realizing a 30% increment in oil/hectare^2, 3^.

Two independent mutations in the *SHELL* gene – a type II MADS-box transcription factor which is homologous with *Arabidopsis* SEEDSTICK (STK) and rice OsMADS13 – were recently reported to be responsible for the fruit forms^4^. The two mutations, *sh* ^*MPOB*^ (T → C, for leucine → proline amino acid change) and *sh* ^*AVROS*^ (A → T, for lysine → asparagine amino acid change) were identified among the descendants of a Nigerian *tenera* accession and a Congo-derived AVROS *pisifera* accession, respectively^4^. These mutations result in amino acid changes within the MADS-box domain and disrupt heterodimerization (*sh* ^*MPOB*^) and DNA binding (*sh* ^*AVROS*^)^4, 5^, respectively. The homozygous wild-type *sh* ^*DeliDura*^ nucleotides at both variant positions are carried by *dura* palms. Palms which are heterozygous for either of the two mutations (s*h* ^*DeliDura*^/*sh* ^*MPOB*^ or *sh* ^*DeliDura*^/*sh* ^*AVROS*^) produce *tenera* palms. *Pisifera* palms are homozygous for either mutation or heteroallelic for both mutations. In other words, the most obvious causative mechanism for the co-dominant fruit form trait is mainly due to haploinsufficiency, wherein only a single functional copy of *SHELL* gene in a diploid genome is insufficient to maintain the wild-type function i.e. *dura*.

From the mass screening of commercial *tenera* materials, another three non-synonymous variants (*sh* ^*MPOB2*^, *sh* ^*MPOB3*^ and *sh* ^*MPOB4*^) within the same MADS-box coding regions controlling the fruit form phenotype in a similar manner were then identified^6^. An example of potential use would be for *pisifera* breeding, where both *pisifera* and *tenera* are assessed, but *dura* discarded. The markers developed could be used to remove the undesired *dura* seeds derived from *tenera* × *tenera* crosses at the pre-nursery stage. This will improve both genetic gain over generations and trial management through reduced land requirement. Also, the *SHELL* genetic testing could potentially increase the oil yield of planted areas by eliminating the lower-yielding, non-*tenera* contaminants prior to field planting^6^. The reported mutations in the *SHELL* gene are applicable in Nigerian and the commercial AVROS accessions, separately. However, some breeding institutes are aiming to broaden the genetic base of AVROS accessions through introgression, or have switched to other genetic resources for paternal lineages, such as Ekona, Yangambi, LaMe and *Serdang* fertile *pisifera* (SP). A better understanding of the heterozygous action of the loss-of-function mutations in *SHELL* gene is also essential to further improve fruit form predictability, especially for introgressive hybrids of oil palm.

## Result and Discussion

**Figure 1:**
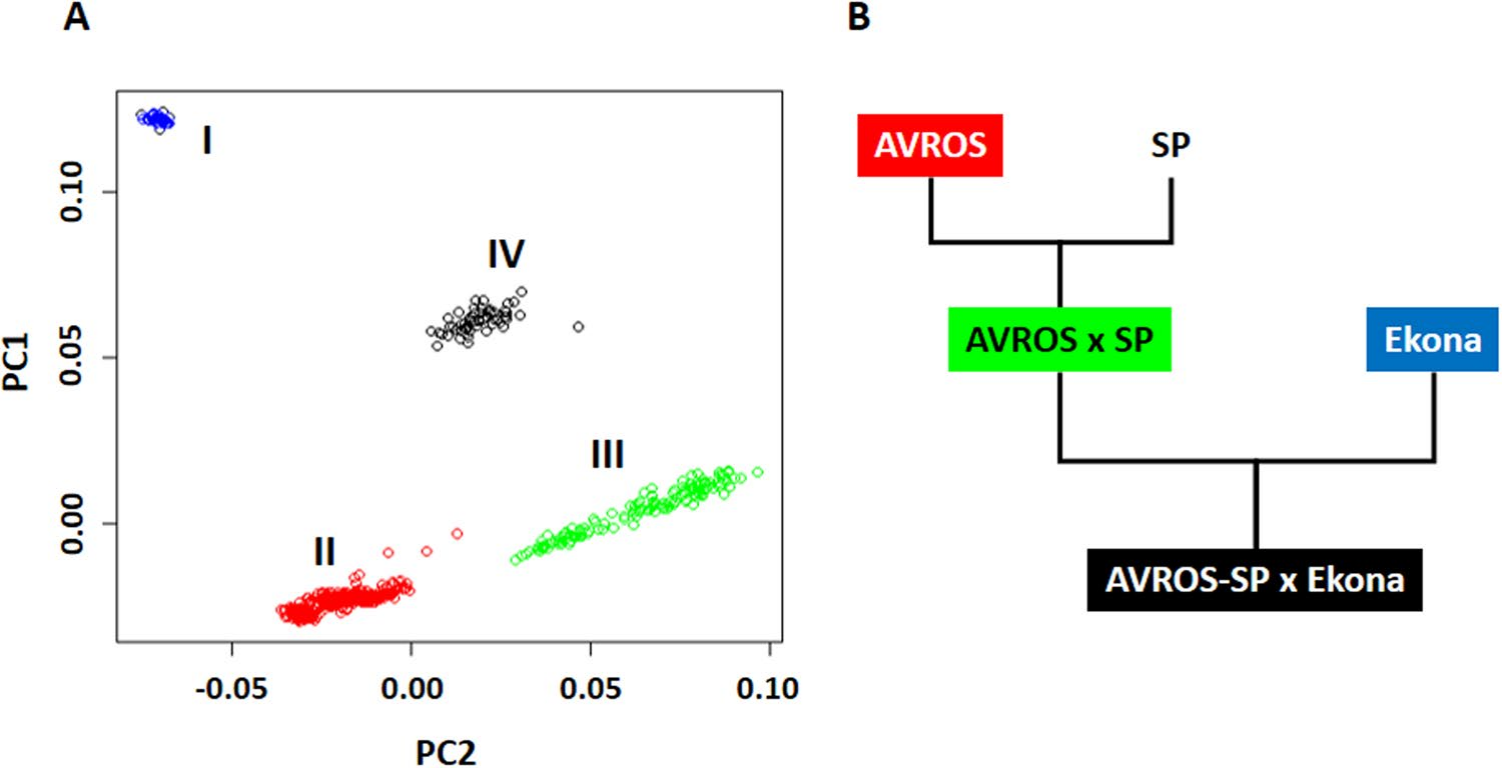
Genetic stratification of 587 oil palm samples, as the first validation set representing four paternal stocks. (**A**) Four clusters i.e. I) Ekona, II) AVROS, III) AVROS x SP, and IV) AVROS-SP × Ekona corresponding to their known origins were detected. PC1 and PC2 indicate the scores of principle components 1 and 2, respectively. (**B**) A pedigree of the four origins. SP – *Serdang* fertile *pisifera*.

In this study, we carried out *SHELL* genetic testing on Sime Darby Plantation’s repertoire of genetic materials using the *sh* ^*MPOB*^ and *sh* ^*AVROS*^ tests. The objectives were to validate the *SHELL* markers on these materials and understand the effect of introgression from different *pisifera* sources on fruit form prediction. As the first phase of validation, three fruit forms (120 *dura*, 305 *tenera* and 162 *pisifera*) belonging to four paternal stocks were selected (Table 1). The genetic stratification of the 587 palms was determined through principal component analysis (PCA) based on our OP200K genotyping array^7^. Four main clusters i.e. I) Ekona, II) AVROS, III) AVROS x SP, IV) AVROS-SP × Ekona (Fig. 1A) were identified and these showed a good agreement with the known pedigree information (Fig. 1B). The AVROS *pisifera* is selected for superiority in growth uniformity, precocity and mesocarp oil content when combined with Deli *dura* origins to produce commercial *tenera* planting materials. However, the narrow genetic base of AVROS (derived from only one palm selected from the self-pollination of the SP540 palm which was again self-pollinated, although there is evidence for some illegitimacy from the final self-pollination) may hinder future breeding progress in oil palm^8^. Oil palm breeders, hence, are actively looking for other genetic resources to enrich the genetic diversity of the current AVROS accession (cluster II). One of these is SP, which was initially developed in the 1960’s for shell-less fertile *pisifera* planting materials^9^, but seed germination and oil extraction from *pisifera* fruits still remains challenging^2^. By having AVROS × SP (cluster III), the breeders aimed to further reduce shell thickness in the commercial *tenera* without complete loss of the shell. Another examined option was the Ekona origin (cluster I) which originated from Cameroon. The trial result showed that Ekona *pisifera* gave smaller trunk height increments, as good or even better bunch production and oil/bunch than the AVROS *pisifera* ^10, 11^. In Sime Darby, we have further introgressed cluster I into cluster III to generate AVROS-SP × Ekona hybrids (cluster IV) (Fig. 1B). This relationship was clearly reflected as cluster IV was located in between cluster I and cluster III (Fig. 1A).

**Table 1:** The first validation set consists of three fruit forms belong to four paternal stocks.

Accession	No. of families	D	T	P	Total
AVROS	25	73	197	84	354
AVROS × SP	15	12	70	58	140
AVROS-SP × Ekona	1	23	24	10	57
Ekona	1	12	14	10	36
Total	42	120	305	162	**587**

SP – *Serdang* fertile *pisifera*; D – *dura*; T – *tenera*; P – *pisifera*.

**Figure 2:**
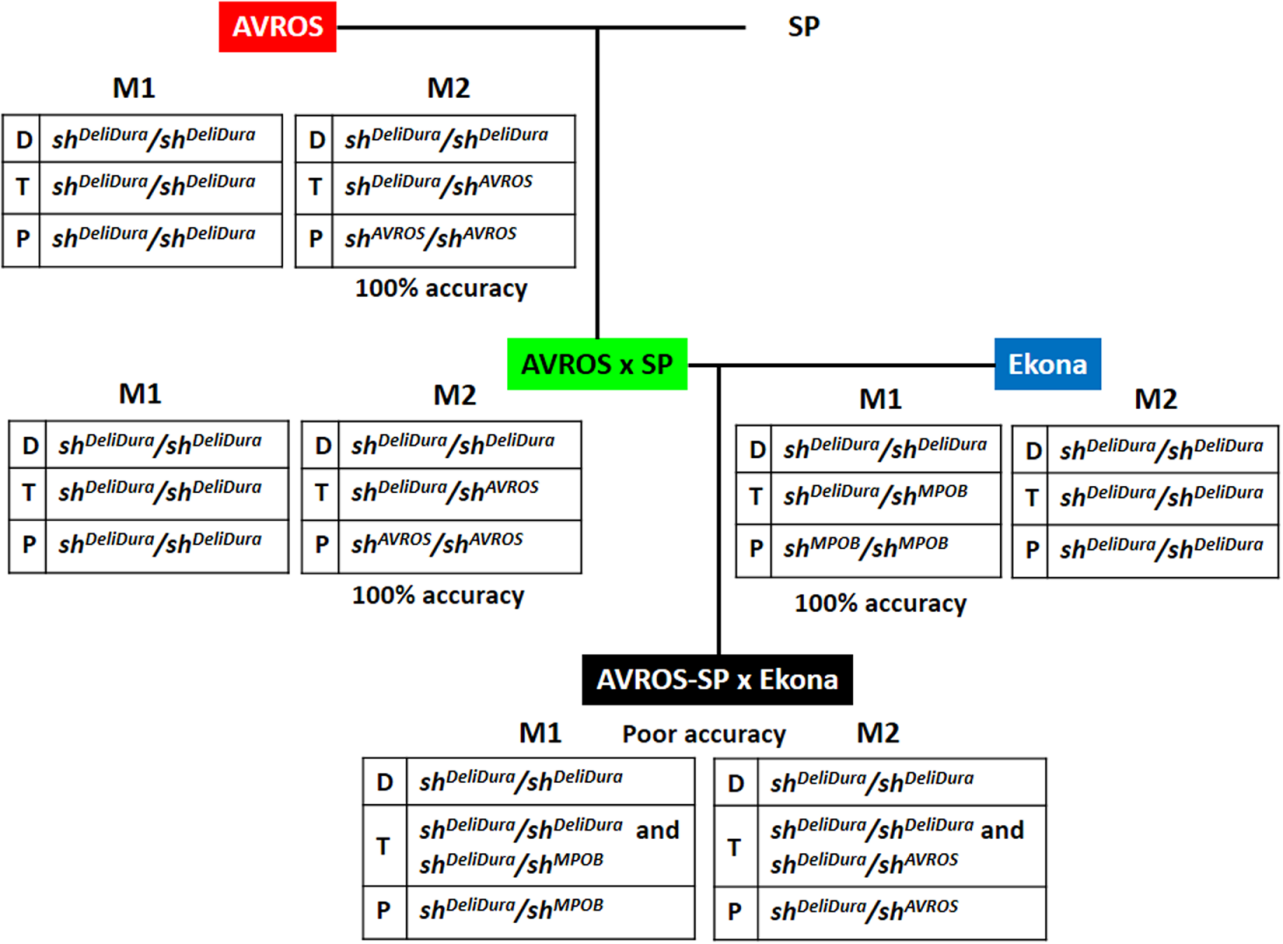
Fruit form prediction using *SHELL* markers in different accessions. Single marker prediction was highly accurate in the accessions tested, except in AVROS-SP (*Serdang* fertile *pisifera*) × Ekona.

The fruit forms of individual palms were phenotyped based on visual inspection of shell thickness and the diagnostic presence of a fiber ring around the kernel in the *tenera* fruit. To validate the accuracy of *SHELL* markers, the fruit form data was compared to the genotype data. The two mutations were defined as M1 for *sh* ^*MPOB*^ and M2 for *sh* ^*AVROS*^, respectively. In general, the prediction accuracy of the *SHELL* markers was successfully validated (100% accuracy), except in the AVROS-SP × Ekona accession (Fig. 2) (Supplementary Table 1). The AVROS accession and the Ekona accession carried *sh* ^AVROS^ and *sh* ^*MPOB*^ mutations, respectively, which may have arisen independently in these accessions derived from central and western Africa. The introgressive hybridization between AVROS × SP and Ekona origins introduced polymorphism for both M1 and M2 (Fig. 2) (Supplementary Table 1) into their progenies. Using single marker prediction, neither M1 nor M2 correctly predicted the segregating *dura*, *tenera* and *pisifera* fruit forms. We then expanded the *SHELL* testing using a second validation set with a total of 752 palms representing AVROS, MPOB, Congo, Tanzanian and four introgressive hybrid origins (AVROS × Nigerian, Cameroon × Congo, Deli-Nigerian × URT-Calabar and URT × Calabar) (Table 2) (Supplementary Table 2). The same result was observed, where the M2 was carried by Congo and Tanzanian originated from central and eastern Africa. The fruit forms of the pure accessions (except AVROS accession with 72.00% of accuracy) thus can be predicted accurately using a single marker, but not in the introgressed hybrids (Table 2) (Supplementary Table 2).

**Table 2:** The prediction accuracy of fruit form based on haploinsufficiency model and genotypes of assayed samples.

Accession	N	Predicted fruit form			Observed fruit form			Observed genotype			Accuracy (%)
		D	T	P	D	T	P	D	T	P	
AVROS	11	3	5	3	2	4	5	(*T*/*T*, *A*/*A*)	(*T*/*T*, *T*/*A*)	(*T*/*T*, *T*/*T*)	72.00
*Nigerian × AVROS	281	69	206	6	69	206	6	(*T*/*T*, *A*/*A*)	(*C*/*T*, *A*/*A*) *&* (*T*/*T*, *T*/*A*)	(*C*/*T*, *T*/*A*), *(C*/*C*, *A*/*A) & (T*/*T*, *T*/*T)*	100.00
*Cameroon × Congo	57	13	29	15	12	29	16	(*T*/*T*, *A*/*A*)	(*C*/*T*, *A*/*A*) *&* (*T*/*T*, *T*/*A*)	(*C*/*T*, *T*/*A*), (*C*/*C*, *A*/*A*) & (*T*/*T*, *T*/*T*)	96.00
*Deli-Nigerian × URT- Calabar	95	11	84	0	11	84	0	(*T*/*T*, *A*/*A*)	(*C*/*T*, *A*/*A*) *&* (*T*/*T*, *T*/*A*)		100.00
*URT × Calabar	1	0	0	1	0	0	1			(*C*/*T*, *T*/*A*)	100.00
MPOB	136	136	0	0	136	0	0	(*T*/*T*, *A*/*A*)			100.00
Tanzanian	131	97	33	1	97	33	1	(*T*/*T*, *A*/*A*)	(*T*/*T*, *T*/*A*)	(*T*/*T*, *T*/*T*)	100.00
Congo	40	14	20	6	14	20	6	(*T*/*T*, *A*/*A*)	(*T*/*T*, *T*/*A*)	(*T*/*T*, *T*/*T*)	100.00

The global accuracy of the second validation set = 99.34%. *Prediction accuracy based on functional copy (haploinsufficiency effect); N – number of samples; D – *dura*; T – *tenera*; P – *pisifera*. For (M1, M2), both *T*/*T* and *A*/*A* are *sh* ^*Deli Dura*^/*sh* ^*Deli Dura*^ as wild type; M1 - *T*/*C* and *C*/*C* are *sh* ^*Deli Dura*^/*sh* ^*MPOB*^ and *sh* ^*MPOB*^/*sh* ^*MPOB*^; M2 - *T*/*A* and *T*/*T* are *sh* ^*Deli Dura*^/*sh* ^*AVROS*^ and *sh* ^*AVROS*^/*sh* ^*AVROS*^.

**Figure 3:**
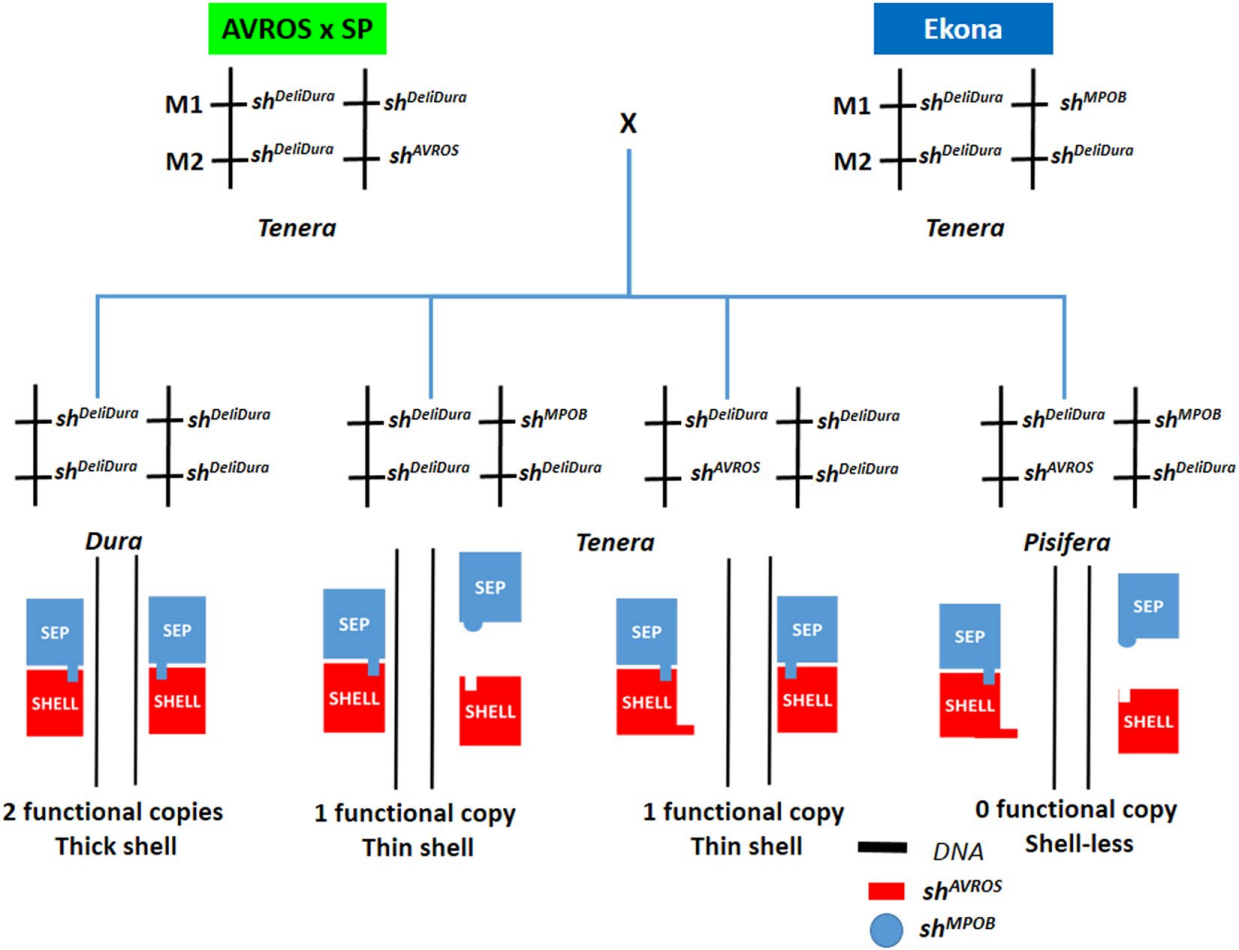
The mapping of genotype-phenotype relationships on an AVROS-SP *tenera* × Ekona *tenera* hybrid population and SHELL-SEP-like protein interaction model in three fruit forms influenced by functional copy (haploinsufficiency effect). The *pisifera* fruit form was due to trans-compound heterozygosity in *SHELL* gene.

To interpret this further, we mapped all the genotype-phenotype relationships onto an AVROS-SP *tenera* × Ekona *tenera* hybrid population (Fig. 3). The AVROS-SP *tenera* containing a single heterozygous mutation at M2 (*sh* ^*DeliDura*^/*sh* ^*DeliDura*^; *sh* ^*DeliDura*^/*sh* ^*AVROS*^) was hybridized to the Ekona *tenera* that carried the other single heterozygous mutation at M1 (*sh* ^*DeliDura*^/*sh* ^*MPOB*^; *sh* ^*DeliDura*^/*sh* ^*DeliDura*^), and the cross produced four genotypes in the hybrid population, according to Mendelian inheritance. In this population, we confirmed the presence of the haploinsufficiency effect on the *SHELL* gene. Not all loss-of-function mutations of an encoded protein are deleterious. Some heterozygous loss-of-function mutations, mainly due to haploinsufficiency, even give selective advantages^12^. The scenario was clearly demonstrated in thin-shelled *tenera*, which produce 30% more oil with full fertility compared to their *dura* and *pisifera* parents. The *tenera* palms in this study contained only a single functional copy of a gene per diploid genome causing an inability to produce enough functional protein as wild-type *dura* palms in the cells; this leads to a haploinsufficiency of the heterodimer which mediates the effects of the *SHELL* gene. Hence, we scored the wild-type *sh* ^*DeliDura*^/*sh* ^*DeliDura*^ which occurred at both M1 and M2 as two functional copies; one functional copy for a single mutation at either the M1 or M2 variant position per diploid; and nil functional copy for multiple mutations at both M1 and M2 positions. Next, we link the haploinsufficiency effect to the shell formation model through SHELL-SEP-like protein heterodimerization and DNA binding^4–6^ (Fig. 3). The *dura* palms with two functional copies were not disrupted in shell formation at all, as expected. As for *tenera*, the two genotypes possible only contributed one functional copy of the SHELL protein, which explained the thinner shell compared to *dura*. One of the alleles led to an inactive protein heterodimer due to failure in either DNA binding for *sh* ^*AVROS*^ carriers, or SHELL-SEP-like dimerization itself for *sh* ^*MPOB*^ carriers. All the shell-less *pisifera* palms were heteroallelic for both mutations (nil functional copy). Therefore, it is important to determine the polymorphism at M1 and M2 first to decide which prediction method should be used. Here, we can observe the clear effect of the causal variants influencing the shell thickness when introgression between certain populations takes place. By shifting single marker analysis to functional copy counting, the prediction accuracies of fruit form in the four introgressive hybrids were significantly improved, ranging from 96.00 to 100.00% (Table 2). Nevertheless, we could not observe any association between both mutations and fertile *pisifera* (with the availability of the four fertile *pisifera*) in AVROS-SP accessions, indicating that the *SHELL* gene itself might not be responsible solely for female sterility in *pisifera* (Supplementary Table 1).

**Figure 4:**
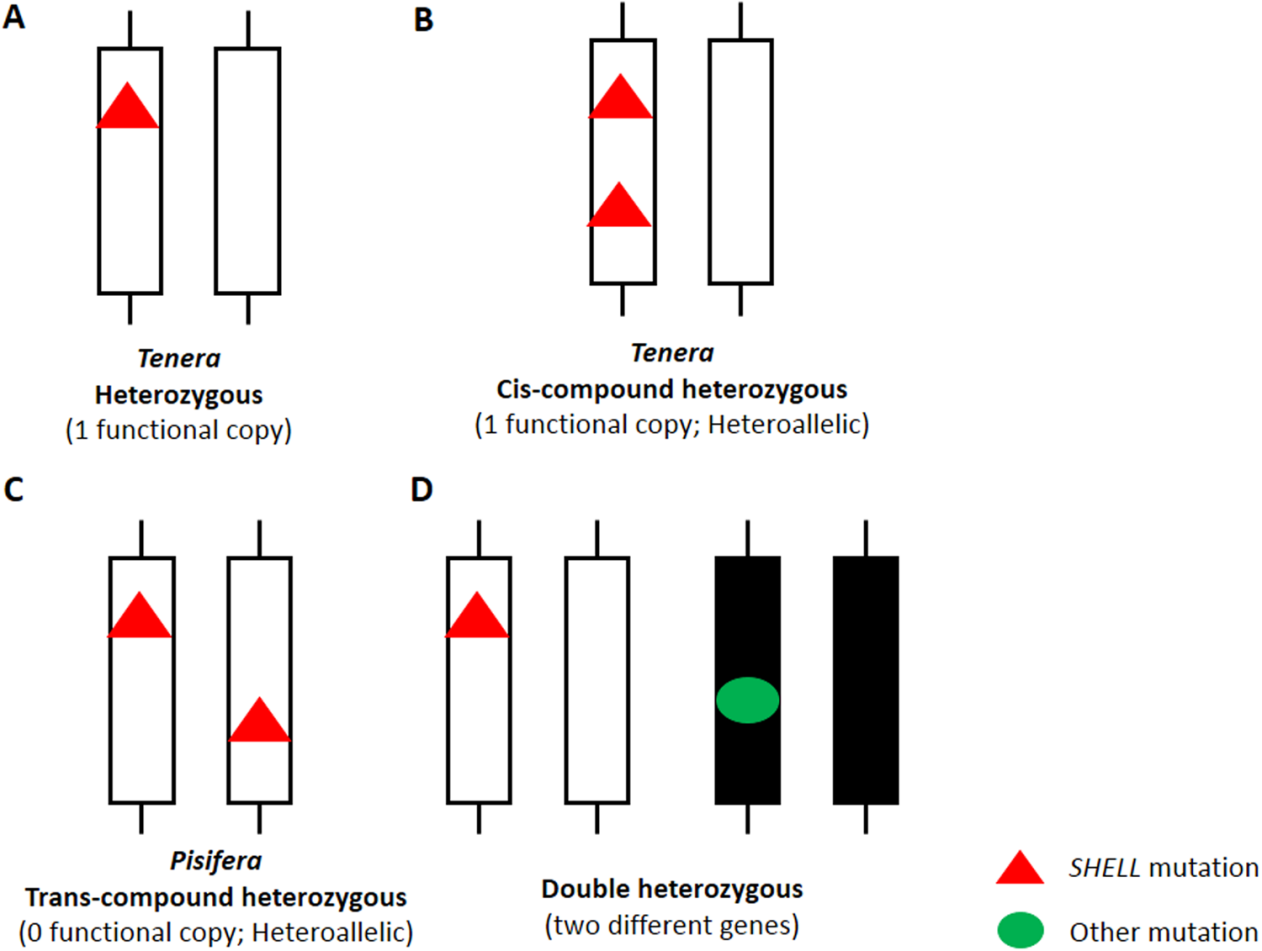
The zygosity of loss-of-function mutations in *SHELL* gene. (**A**) *Tenera* palm that carried a single heterozygous mutation, only produce 1 functional copy of the gene. (**B**) Cis-compound heterozygous *tenera* palms produce 1 functional copy, when both mutations occur on the same chromosome. (**C**) Trans-compound heterozygous *pisifera* palms produce 0 functional copy, when both mutations occur on different chromosome. (**D**) In double heterozygous individuals, the mutations occurred in different genes.

The global accuracy in the second validation set was extremely high (99.34%), but unexpected low accuracy (72.00%) in one AVROS accession drew our attention (Table 2). The phenotype-genotype discrepancies might be due to the presence of other novel mutants on the *SHELL* gene, as reported^6^. Hence, a total of 13 palms (5 phenotype-genotype discrepancies and 8 controls) were tested using all five reported mutant *SHELL* alleles including *sh* ^*AVROS*^, *sh* ^*MPOB*^, *sh* ^*MPOB2*^, *sh* ^*MPOB3*^ and *sh* ^*MPOB4*^ within the MADS-box domain. The three AVROS discrepancies were successfully rectified based on *sh* ^*MPOB2*^ and *sh* ^*AVROS*^ mutations (Table 3), achieving 100.00% prediction accuracy in the accession. However, this did not apply to two Cameroon × Congo discrepancies, indicating probable occurrence of other novel mutations (Table 3). We thus expect another heterozygous mutation for V599 *pisifera* and V927 *tenera*. Interestingly, we also discovered a heteroallelic case for three mutations in V121 *pisifera* in Nigerian × AVROS accession. This implied that some accessions/origins can possess more than two mutations or a different cluster of mutations, although it can be rare. However, this will become prominent during introgression of wild germplasm. The compound mutation in the *SHELL* gene for fruit form phenotype in oil palm was firstly discussed in this study. Multiple mutations that occur in the same gene and in different genes, are defined as compound mutation and double mutation, respectively^13, 14^ (Fig. 4). All the reported mutations are located in the *SHELL* gene, therefore known as compound mutations which can be heterozygous or homozygous. The loss-of-function compound mutations can happen in cis (mutations on the same chromosome) (Fig. 4B) and trans forms (mutations on the different chromosome) (Fig. 4C) too. To date, the haploinsufficient *SHELL* gene for *pisifera* is mainly explained by trans-compound heterozygous mutations only, particularly in introgressive hybrids. Nevertheless, we should not ignore the possible existence of cis-compound heterozygous palms that are still able to produce one functional copy to express the *tenera* fruit form (Fig. 4B). Hence, these individual palms can be misinterpreted as *pisifera* if based on heteroallelic tests for both mutations only.

**Table 3:** The prediction accuracy of fruit form based on haploinsufficiency model and genotypes of five discrepancies and eight controls tested in the second validation set.

Sample ID	Accession	*Sh* ^*MPOB2*^	*Sh* ^*MPOB4*^	*Sh* ^*MPOB*^	*Sh* ^*AVROS*^	*Sh* ^*MPOB3*^	Predicted Fruit Form	Observed Fruit Form	Accuracy
V907	*Cameroon × Congo	*A*/*A*	*A*/*A*	*T*/*T*	*A*/*A*	*C*/*C*	D	D	Concordant
V670	*Congo	*A*/*A*	*A*/*A*	*T*/*T*	*A*/*A*	*C*/*C*	D	D	Concordant
V749	*MPOB	*A*/*A*	*A*/*A*	*T*/*T*	*A*/*A*	*C*/*C*	D	D	Concordant
V121	*Nigerian × AVROS	*A*/*A*	*T*/A**	*C*/T**	*T*/A**	*C*/*C*	P	P	Concordant
V152	*Tanzanian	*A*/*A*	*A*/*A*	*T*/*T*	*T*/T***	*C*/*C*	P	P	Concordant
V685	*Congo	*A*/*A*	*A*/*A*	*T*/*T*	*T*/T***	*C*/*C*	P	P	Concordant
V646	*Congo	*A*/*A*	*A*/*A*	*T*/*T*	*T*/A**	*C*/*C*	T	T	Concordant
V086	*Nigerian × AVROS	*A*/*A*	*A*/*A*	*C*/T**	*A*/*A*	*C*/*C*	T	T	Concordant
C2	AVROS	*C*/A**	*A*/*A*	*T*/*T*	*A*/*A*	*C*/*C*	T	T	Corrected
C1	AVROS	*C*/A**	*A*/*A*	*T*/*T*	*T*/A**	*C*/*C*	P	P	Corrected
C3	AVROS	*C*/A**	*A*/*A*	*T*/*T*	*T*/A**	*C*/*C*	P	P	Corrected
V927	Cameroon × Congo	*A*/*A*	*A*/*A*	*T*/*T*	*A*/*A*	*C*/*C*	D	T	Miss
V599	Cameroon × Congo	*A*/*A*	*A*/*A*	*C*/*T* ^*a*^	*A*/*A*	*C*/*C*	T	P	Miss

*Control samples tested using M1 and M2 in the second validation set. D – *dura*; T – *tenera*; P – *pisifera*. Highlighted genotype indicated the presence of mutation (**heterozygous; ***homozygous).

In conclusion, the haploinsufficiency effect on *SHELL* gene responsible for fruit form variation in oil palm was further characterized based on identity-by-descent. The reported *sh* ^*AVROS*^, *sh* ^*MPOB*^, *sh* ^*MPOB2*^, *sh* ^*MPOB3*^ and *sh* ^*MPOB4*^ within MADS-box domain should be adopted together to improve prediction accuracy, especially in the introgressive hybrid materials. Furthermore, the existence of cis-compound heterozygous palms should be confirmed through continuous screening of various germplasm. This study provides a good reference for other crop and animal breeding programs where the understanding of causative variant segregation is extremely important, even for monogenic traits such as oil palm shell thickness.

## Methods

### Sampling and DNA Preparation

A total of 1,339 oil palm samples were selected and divided into two validation sets. The first validation set included 587 palms representing four different accessions, i.e. AVROS, AVROS × SP, AVROS-SP × Ekona and Ekona maintained at Sime Darby Plantation R&D Centre in Malaysia (Table 1). The validation set consisted of three different fruit forms (thick-shelled *dura*, thin-shelled *tenera* and shell-less *pisifera*). The pedigree was provided by the breeders as indicated in Fig. 1B. Subsequently, the study was extended to the second validation set derived from more diverse backgrounds, also with a larger overall group size (752 palms) (Table 2). The background included four pure accessions (AVROS, MPOB, Tanzanian and Congo) and four introgressed hybrid populations (Nigerian × AVROS, Cameroon × Congo, Deli-Nigerian × URT Calabar and URT × Calabar). Total genomic DNA was isolated from 0.1 g of young leaf tissue (frond 0) using the DNAeasy Plant Mini Kit (Qiagen).

### Genetic stratification analysis

To understand the genetic structure, the first validation set was genotyped using genome-wide SNP markers on a OP200K Infinium array^7^ (Illumina). The genotypic data of 200,000 SNPs were filtered based on minor allele frequency (MAF) >0.01 and >90% call rate. Subsequently, the genetic stratification among 586 oil palm samples was analyzed in the R package SNPRelate^15^, using the default parameters.

### *SHELL* genotyping

About 3 ng of genomic DNA was used as template for both specific allele tests. The probe design for *sh* ^*MPOB*^, *sh* ^*AVROS*^, *sh* ^*MPOB2*^, *sh* ^*MPOB3*^ and *sh* ^*MPOB4*^ mutations was done at their reported genomic positions: 3,078,161 bp, 3,078,154 bp, 3,078,180 bp, 3,078,125 bp and 3,078,178 bp on Chromosome 2, namely CM002082.1^4, 6^ based on the requirement of Kompetitive Allele Specific PCR^TM^ (KASP^TM^) genotyping platform. Two fluorophores FAM and HEX were used to distinguish the KASP^TM^ genotyping data. In the clustering plot, the samples marked red were homozygous for alleles reported with HEX, whereas those marked blue were homozygous for FAM allele. The heterozygous samples appear as green.

### Genotype-phenotype analysis

The fruit form census was performed based on visual examination of shell thickness and the presence or absence of a fiber ring in each cross-sectioned fruitlet. In addition, fertility status of AVROS-SP *pisifera* was also recorded based on the presence of embryo in the palm kernel. The observed fruit form and fertility data were then compared to the predicted data based on the reported *sh* ^MPOB^ and *sh* ^*AVROS*^ variants^4^. From there, accuracy (in %) of the single marker and haploinsufficiency models in each accession, except 15 samples with missing data were evaluated (Supplementary Table 1). The single marker method predicted the fruit forms based on either *sh* ^MPOB^ or *sh* ^*AVROS*^ variant. But, the haploinsufficiency model considered both variants simultaneously. The wild-type *sh* ^*DeliDura*^ occurred at both M1 and M2 per chromosome, with 2 functional copies of the M1-M2 haplotype for *dura*; a single mutation at either M1 or M2 per chromosome as 1 functional copy for *tenera*; and compound mutations at M1 and M2 on both haplotypes as 0 functional copy for *pisifera*. The analysis was then extended to another three mutations, including *sh* ^*MPOB2*^, *sh* ^*MPOB3*^ and *sh* ^*MPOB4*^ tested on five discrepancies and eight controls based on the same haploinsufficiency model.

## Electronic supplementary material

Supplementary Information 
