# Supplementary material for: Characterizing haploinsufficiency of SHELL gene to improve fruit form prediction in introgressive hybrids of oil palm
Source: Sci Rep. 2017 Jun 8;7:3118. doi: 10.1038/s41598-017-03225-7 (PMC5465187; doi:10.1038/s41598-017-03225-7)
Supplement: Supplementary file 1 — Supplementary Information [file 41598_2017_3225_MOESM1_ESM.pdf]

Characterizing haploinsufficiency of the *SHELL* gene to improve fruit form prediction in introgressive hybrids of oil palm

Chee-Keng Teh[1], Siti Dalila Muaz[1], Praveena Tangaya[1], Po-Yee Fong[1], Ai-Ling Ong[1], Sean Mayes[2], Fook-Tim Chew[3], Harikrishna Kulaveerasingam[1] & David Appleton[1]

[1]Biotechnology & Breeding Department, Sime Darby Plantation R&D Centre, Malaysia.

[2]School of Biosciences, University of Nottingham, UK.

[3]Department of Biological Sciences, National University of Singapore, Singapore.

Correspondence should be addressed to: C.T. (teh.chee.keng@simedarby.com)

Supplementary Materials

Index

Supplementary Table 1. The genotype-phenotype analysis of fruit form in the first validation set based on single marker and genotypes of assayed samples.

Supplementary Table 2. The genotype-phenotype analysis of fruit form in the second validation set based on haploinsufficiency model and genotypes of assayed samples.

Supplementary Table 1. The genotype-phenotype analysis of fruit form the first validation set based on single marker and genotypes of assayed samples.

Sample ID	Accession	Sh^{MPOB}	Sh^{AVROS}	Predicted Fruit Form (Single Marker)	Observed Fruit Form	Accuracy
C1026	AVROS	T/T	*A/A*	D	D	
C1031	AVROS	T/T	*A/A*	D	D	
C1033	AVROS	T/T	*A/A*	D	D	
C1035	AVROS	T/T	*A/A*	D	D	
C1036	AVROS	T/T	*A/A*	D	D	
C1038	AVROS	T/T	*A/A*	D	D	
C1045	AVROS	T/T	*A/A*	D	D	
C1046	AVROS	T/T	*A/A*	D	D	
C1048	AVROS	T/T	*A/A*	D	D	
C1049	AVROS	T/T	*A/A*	D	D	
C1050	AVROS	T/T	*A/A*	D	D	
C1051	AVROS	T/T	*A/A*	D	D	
C1061	AVROS	T/T	*A/A*	D	D	
C1065	AVROS	T/T	*A/A*	D	D	
C1068	AVROS	T/T	*A/A*	D	D	
C1072	AVROS	T/T	*A/A*	D	D	
C1191	AVROS	T/T	*A/A*	D	D	
C1214	AVROS	T/T	*A/A*	D	D	
C1215	AVROS	T/T	*A/A*	D	D	
C1216	AVROS	T/T	*A/A*	D	D	
C1220	AVROS	T/T	*A/A*	D	D	
C2222	AVROS	T/T	*A/A*	D	D	
C2223	AVROS	T/T	*A/A*	D	D	
C2224	AVROS	T/T	*A/A*	D	D	
C2231	AVROS	T/T	*A/A*	D	D	
C2235	AVROS	T/T	*A/A*	D	D	
C2240	AVROS	T/T	*A/A*	D	D	
C2262	AVROS	T/T	*A/A*	D	D	
C2263	AVROS	T/T	*A/A*	D	D	
C2268	AVROS	T/T	*A/A*	D	D	
C2277	AVROS	T/T	*A/A*	D	D	
C2280	AVROS	T/T	*A/A*	D	D	
C2287	AVROS	T/T	*A/A*	D	D	
C2290	AVROS	T/T	*A/A*	D	D	
C2292	AVROS	T/T	*A/A*	D	D	
C2301	AVROS	T/T	*A/A*	D	D	
C2305	AVROS	T/T	*A/A*	D	D	
C2306	AVROS	T/T	*A/A*	D	D	

C2316	AVROS	T/T	*A/A*	D	D
C2320	AVROS	T/T	*A/A*	D	D
C2321	AVROS	T/T	*A/A*	D	D
C2328	AVROS	T/T	*A/A*	D	D
C2331	AVROS	T/T	*A/A*	D	D
C2332	AVROS	T/T	*A/A*	D	D
C2337	AVROS	T/T	*A/A*	D	D
C2339	AVROS	T/T	*A/A*	D	D
C2342	AVROS	T/T	*A/A*	D	D
C2343	AVROS	T/T	*A/A*	D	D
C2348	AVROS	T/T	*A/A*	D	D
C2354	AVROS	T/T	*A/A*	D	D
C2357	AVROS	T/T	*A/A*	D	D
C2358	AVROS	T/T	*A/A*	D	D
C2364	AVROS	T/T	*A/A*	D	D
C2374	AVROS	T/T	*A/A*	D	D
C238	AVROS	T/T	*A/A*	D	D
C2412	AVROS	T/T	*A/A*	D	D
C2414	AVROS	T/T	*A/A*	D	D
C2423	AVROS	T/T	*A/A*	D	D
C2425	AVROS	T/T	*A/A*	D	D
C2426	AVROS	T/T	*A/A*	D	D
C2431	AVROS	T/T	*A/A*	D	D
C2435	AVROS	T/T	*A/A*	D	D
C2436	AVROS	T/T	*A/A*	D	D
C2441	AVROS	T/T	*A/A*	D	D
C2442	AVROS	T/T	*A/A*	D	D
C2444	AVROS	T/T	*A/A*	D	D
C275	AVROS	T/T	*A/A*	D	D
C276	AVROS	T/T	*A/A*	D	D
C294	AVROS	T/T	*A/A*	D	D
C333	AVROS	T/T	*A/A*	D	D
C350	AVROS	T/T	*A/A*	D	D
C1029	AVROS	0/0	*0/0*	Excluded	D
C1039	AVROS	0/0	*0/0*	Excluded	T
C2258	AVROS	T/T	*0/0*	Excluded	T
C2335	AVROS	T/T	*0/0*	Excluded	P
C2385	AVROS	0/0	*T/T*	Excluded	P
C2451	AVROS	T/T	*0/0*	Excluded	P
C2841	AVROS	0/0	*A/A*	Excluded	D
C1000	AVROS	T/T	*T/T*	P	P
C1004	AVROS	T/T	*T/T*	P	P
C1005	AVROS	T/T	*T/T*	P	P

C1006	AVROS	T/T	*T/T*	P	P
C1008	AVROS	T/T	*T/T*	P	P
C1012	AVROS	T/T	*T/T*	P	P
C1017	AVROS	T/T	*T/T*	P	P
C1018	AVROS	T/T	*T/T*	P	P
C1019	AVROS	T/T	*T/T*	P	P
C1022	AVROS	T/T	*T/T*	P	P
C1028	AVROS	T/T	*T/T*	P	P
C1044	AVROS	T/T	*T/T*	P	P
C1052	AVROS	T/T	*T/T*	P	P
C1053	AVROS	T/T	*T/T*	P	P
C1063	AVROS	T/T	*T/T*	P	P
C1067	AVROS	T/T	*T/T*	P	P
C1074	AVROS	T/T	*T/T*	P	P
C1075	AVROS	T/T	*T/T*	P	P
C1076	AVROS	T/T	*T/T*	P	P
C1080	AVROS	T/T	*T/T*	P	P
C1082	AVROS	T/T	*T/T*	P	P
C1083	AVROS	T/T	*T/T*	P	P
C1189	AVROS	T/T	*T/T*	P	P
C1194	AVROS	T/T	*T/T*	P	P
C1197	AVROS	T/T	*T/T*	P	P
C1198	AVROS	T/T	*T/T*	P	P
C1199	AVROS	T/T	*T/T*	P	P
C1200	AVROS	T/T	*T/T*	P	P
C1201	AVROS	T/T	*T/T*	P	P
C1207	AVROS	T/T	*T/T*	P	P
C1217	AVROS	T/T	*T/T*	P	P
C2220	AVROS	T/T	*T/T*	P	P
C2229	AVROS	T/T	*T/T*	P	P
C2246	AVROS	T/T	*T/T*	P	P
C2250	AVROS	T/T	*T/T*	P	P
C2256	AVROS	T/T	*T/T*	P	P
C2257	AVROS	T/T	*T/T*	P	P
C2265	AVROS	T/T	*T/T*	P	P
C2272	AVROS	T/T	*T/T*	P	P
C2274	AVROS	T/T	*T/T*	P	P
C2285	AVROS	T/T	*T/T*	P	P
C2288	AVROS	T/T	*T/T*	P	P
C2293	AVROS	T/T	*T/T*	P	P
C2294	AVROS	T/T	*T/T*	P	P
C2295	AVROS	T/T	*T/T*	P	P
C2304	AVROS	T/T	*T/T*	P	P

C2309	AVROS	T/T	*T/T*	P	P
C2311	AVROS	T/T	*T/T*	P	P
C2317	AVROS	T/T	*T/T*	P	P
C2322	AVROS	T/T	*T/T*	P	P
C2326	AVROS	T/T	*T/T*	P	P
C2333	AVROS	T/T	*T/T*	P	P
C2341	AVROS	T/T	*T/T*	P	P
C2372	AVROS	T/T	*T/T*	P	P
C2376	AVROS	T/T	*T/T*	P	P
C2377	AVROS	T/T	*T/T*	P	P
C2378	AVROS	T/T	*T/T*	P	P
C2381	AVROS	T/T	*T/T*	P	P
C2395	AVROS	T/T	*T/T*	P	P
C2400	AVROS	T/T	*T/T*	P	P
C2416	AVROS	T/T	*T/T*	P	P
C2419	AVROS	T/T	*T/T*	P	P
C2422	AVROS	T/T	*T/T*	P	P
C2428	AVROS	T/T	*T/T*	P	P
C2429	AVROS	T/T	*T/T*	P	P
C2432	AVROS	T/T	*T/T*	P	P
C2433	AVROS	T/T	*T/T*	P	P
C2438	AVROS	T/T	*T/T*	P	P
C2439	AVROS	T/T	*T/T*	P	P
C2445	AVROS	T/T	*T/T*	P	P
C2450	AVROS	T/T	*T/T*	P	P
C2452	AVROS	T/T	*T/T*	P	P
C256	AVROS	T/T	*T/T*	P	P
C2692	AVROS	T/T	*T/T*	P	P
C274	AVROS	T/T	*T/T*	P	P
C293	AVROS	T/T	*T/T*	P	P
C352	AVROS	T/T	*T/T*	P	P
C354	AVROS	T/T	*T/T*	P	P
C356	AVROS	T/T	*T/T*	P	P
C995	AVROS	T/T	*T/T*	P	P
C997	AVROS	T/T	*T/T*	P	P
C1001	AVROS	T/T	*T/A*	T	T
C1002	AVROS	T/T	*T/A*	T	T
C1003	AVROS	T/T	*T/A*	T	T
C1007	AVROS	T/T	*T/A*	T	T
C1009	AVROS	T/T	*T/A*	T	T
C1010	AVROS	T/T	*T/A*	T	T
C1011	AVROS	T/T	*T/A*	T	T
C1013	AVROS	T/T	*T/A*	T	T

C1014	AVROS	T/T	*T/A*	T	T
C1015	AVROS	T/T	*T/A*	T	T
C1016	AVROS	T/T	*T/A*	T	T
C1020	AVROS	T/T	*T/A*	T	T
C1021	AVROS	T/T	*T/A*	T	T
C1023	AVROS	T/T	*T/A*	T	T
C1024	AVROS	T/T	*T/A*	T	T
C1025	AVROS	T/T	*T/A*	T	T
C1027	AVROS	T/T	*T/A*	T	T
C1030	AVROS	T/T	*T/A*	T	T
C1032	AVROS	T/T	*T/A*	T	T
C1034	AVROS	T/T	*T/A*	T	T
C1037	AVROS	T/T	*T/A*	T	T
C1040	AVROS	T/T	*T/A*	T	T
C1041	AVROS	T/T	*T/A*	T	T
C1042	AVROS	T/T	*T/A*	T	T
C1043	AVROS	T/T	*T/A*	T	T
C1047	AVROS	T/T	*T/A*	T	T
C1054	AVROS	T/T	*T/A*	T	T
C1055	AVROS	T/T	*T/A*	T	T
C1056	AVROS	T/T	*T/A*	T	T
C1057	AVROS	T/T	*T/A*	T	T
C1059	AVROS	T/T	*T/A*	T	T
C1064	AVROS	T/T	*T/A*	T	T
C1066	AVROS	T/T	*T/A*	T	T
C1071	AVROS	T/T	*T/A*	T	T
C1073	AVROS	T/T	*T/A*	T	T
C1078	AVROS	T/T	*T/A*	T	T
C1079	AVROS	T/T	*T/A*	T	T
C1081	AVROS	T/T	*T/A*	T	T
C1084	AVROS	T/T	*T/A*	T	T
C1085	AVROS	T/T	*T/A*	T	T
C1086	AVROS	T/T	*T/A*	T	T
C1087	AVROS	T/T	*T/A*	T	T
C1088	AVROS	T/T	*T/A*	T	T
C1190	AVROS	T/T	*T/A*	T	T
C1193	AVROS	T/T	*T/A*	T	T
C1202	AVROS	T/T	*T/A*	T	T
C1204	AVROS	T/T	*T/A*	T	T
C1205	AVROS	T/T	*T/A*	T	T
C1206	AVROS	T/T	*T/A*	T	T
C1208	AVROS	T/T	*T/A*	T	T
C1210	AVROS	T/T	*T/A*	T	T

C1211	AVROS	T/T	*T/A*	T	T
C1219	AVROS	T/T	*T/A*	T	T
C2221	AVROS	T/T	*T/A*	T	T
C2225	AVROS	T/T	*T/A*	T	T
C2226	AVROS	T/T	*T/A*	T	T
C2227	AVROS	T/T	*T/A*	T	T
C2228	AVROS	T/T	*T/A*	T	T
C2230	AVROS	T/T	*T/A*	T	T
C2232	AVROS	T/T	*T/A*	T	T
C2233	AVROS	T/T	*T/A*	T	T
C2234	AVROS	T/T	*T/A*	T	T
C2236	AVROS	T/T	*T/A*	T	T
C2237	AVROS	T/T	*T/A*	T	T
C2239	AVROS	T/T	*T/A*	T	T
C2241	AVROS	T/T	*T/A*	T	T
C2242	AVROS	T/T	*T/A*	T	T
C2243	AVROS	T/T	*T/A*	T	T
C2244	AVROS	T/T	*T/A*	T	T
C2245	AVROS	T/T	*T/A*	T	T
C2247	AVROS	T/T	*T/A*	T	T
C2248	AVROS	T/T	*T/A*	T	T
C2249	AVROS	T/T	*T/A*	T	T
C2251	AVROS	T/T	*T/A*	T	T
C2252	AVROS	T/T	*T/A*	T	T
C2253	AVROS	T/T	*T/A*	T	T
C2254	AVROS	T/T	*T/A*	T	T
C2255	AVROS	T/T	*T/A*	T	T
C2259	AVROS	T/T	*T/A*	T	T
C2260	AVROS	T/T	*T/A*	T	T
C2261	AVROS	T/T	*T/A*	T	T
C2264	AVROS	T/T	*T/A*	T	T
C2266	AVROS	T/T	*T/A*	T	T
C2267	AVROS	T/T	*T/A*	T	T
C2270	AVROS	T/T	*T/A*	T	T
C2273	AVROS	T/T	*T/A*	T	T
C2276	AVROS	T/T	*T/A*	T	T
C2278	AVROS	T/T	*T/A*	T	T
C2279	AVROS	T/T	*T/A*	T	T
C2281	AVROS	T/T	*T/A*	T	T
C2282	AVROS	T/T	*T/A*	T	T
C2283	AVROS	T/T	*T/A*	T	T
C2284	AVROS	T/T	*T/A*	T	T
C2286	AVROS	T/T	*T/A*	T	T

C2289	AVROS	T/T	*T/A*	T	T
C2291	AVROS	T/T	*T/A*	T	T
C2296	AVROS	T/T	*T/A*	T	T
C2297	AVROS	T/T	*T/A*	T	T
C2298	AVROS	T/T	*T/A*	T	T
C2299	AVROS	T/T	*T/A*	T	T
C2300	AVROS	T/T	*T/A*	T	T
C2302	AVROS	T/T	*T/A*	T	T
C2303	AVROS	T/T	*T/A*	T	T
C2307	AVROS	T/T	*T/A*	T	T
C2308	AVROS	T/T	*T/A*	T	T
C2310	AVROS	T/T	*T/A*	T	T
C2312	AVROS	T/T	*T/A*	T	T
C2313	AVROS	T/T	*T/A*	T	T
C2314	AVROS	T/T	*T/A*	T	T
C2315	AVROS	T/T	*T/A*	T	T
C2318	AVROS	T/T	*T/A*	T	T
C2323	AVROS	T/T	*T/A*	T	T
C2324	AVROS	T/T	*T/A*	T	T
C2325	AVROS	T/T	*T/A*	T	T
C2327	AVROS	T/T	*T/A*	T	T
C2330	AVROS	T/T	*T/A*	T	T
C2334	AVROS	T/T	*T/A*	T	T
C2336	AVROS	T/T	*T/A*	T	T
C2338	AVROS	T/T	*T/A*	T	T
C2340	AVROS	T/T	*T/A*	T	T
C2347	AVROS	T/T	*T/A*	T	T
C2349	AVROS	T/T	*T/A*	T	T
C2350	AVROS	T/T	*T/A*	T	T
C2351	AVROS	T/T	*T/A*	T	T
C2353	AVROS	T/T	*T/A*	T	T
C2355	AVROS	T/T	*T/A*	T	T
C2360	AVROS	T/T	*T/A*	T	T
C2368	AVROS	T/T	*T/A*	T	T
C237	AVROS	T/T	*T/A*	T	T
C2370	AVROS	T/T	*T/A*	T	T
C2371	AVROS	T/T	*T/A*	T	T
C2375	AVROS	T/T	*T/A*	T	T
C2379	AVROS	T/T	*T/A*	T	T
C2380	AVROS	T/T	*T/A*	T	T
C2382	AVROS	T/T	*T/A*	T	T
C2384	AVROS	T/T	*T/A*	T	T
C2386	AVROS	T/T	*T/A*	T	T

C2387	AVROS	T/T	*T/A*	T	T
C2389	AVROS	T/T	*T/A*	T	T
C239	AVROS	T/T	*T/A*	T	T
C2390	AVROS	T/T	*T/A*	T	T
C2391	AVROS	T/T	*T/A*	T	T
C2392	AVROS	T/T	*T/A*	T	T
C2393	AVROS	T/T	*T/A*	T	T
C2394	AVROS	T/T	*T/A*	T	T
C2396	AVROS	T/T	*T/A*	T	T
C2397	AVROS	T/T	*T/A*	T	T
C2398	AVROS	T/T	*T/A*	T	T
C2399	AVROS	T/T	*T/A*	T	T
C2401	AVROS	T/T	*T/A*	T	T
C2402	AVROS	T/T	*T/A*	T	T
C2403	AVROS	T/T	*T/A*	T	T
C2404	AVROS	T/T	*T/A*	T	T
C2405	AVROS	T/T	*T/A*	T	T
C2406	AVROS	T/T	*T/A*	T	T
C2407	AVROS	T/T	*T/A*	T	T
C2408	AVROS	T/T	*T/A*	T	T
C2409	AVROS	T/T	*T/A*	T	T
C2410	AVROS	T/T	*T/A*	T	T
C2411	AVROS	T/T	*T/A*	T	T
C2413	AVROS	T/T	*T/A*	T	T
C2415	AVROS	T/T	*T/A*	T	T
C2417	AVROS	T/T	*T/A*	T	T
C2418	AVROS	T/T	*T/A*	T	T
C2420	AVROS	T/T	*T/A*	T	T
C2421	AVROS	T/T	*T/A*	T	T
C2424	AVROS	T/T	*T/A*	T	T
C2427	AVROS	T/T	*T/A*	T	T
C2430	AVROS	T/T	*T/A*	T	T
C2446	AVROS	T/T	*T/A*	T	T
C2447	AVROS	T/T	*T/A*	T	T
C2448	AVROS	T/T	*T/A*	T	T
C2453	AVROS	T/T	*T/A*	T	T
C253	AVROS	T/T	*T/A*	T	T
C273	AVROS	T/T	*T/A*	T	T
C312	AVROS	T/T	*T/A*	T	T
C313	AVROS	T/T	*T/A*	T	T
C314	AVROS	T/T	*T/A*	T	T
C315	AVROS	T/T	*T/A*	T	T
C349	AVROS	T/T	*T/A*	T	T

C351	AVROS	T/T	*T/A*	T	T
C353	AVROS	T/T	*T/A*	T	T
C355	AVROS	T/T	*T/A*	T	T
C357	AVROS	T/T	*T/A*	T	T
C358	AVROS	T/T	*T/A*	T	T
C379	AVROS	T/T	*T/A*	T	T
C380	AVROS	T/T	*T/A*	T	T
C733	AVROS	T/T	*T/A*	T	T
C734	AVROS	T/T	*T/A*	T	T
C735	AVROS	T/T	*T/A*	T	T
C994	AVROS	T/T	*T/A*	T	T
C996	AVROS	T/T	*T/A*	T	T
C998	AVROS	T/T	*T/A*	T	T
C999	AVROS	T/T	*T/A*	T	T
C2570	AVROS x SP	T/T	*A/A*	D	D
C2580	AVROS x SP	T/T	*A/A*	D	D
C2712	AVROS x SP	T/T	*A/A*	D	D
C2715	AVROS x SP	T/T	*A/A*	D	D
C2717	AVROS x SP	T/T	*A/A*	D	D
C2718	AVROS x SP	T/T	*A/A*	D	D
C2724	AVROS x SP	T/T	*A/A*	D	D
C2865	AVROS x SP	T/T	*A/A*	D	D
C2869	AVROS x SP	T/T	*A/A*	D	D
C2872	AVROS x SP	T/T	*A/A*	D	D
C2874	AVROS x SP	T/T	*A/A*	D	D
C2501	AVROS x SP	0/0	*0/0*	Excluded	T
C2511	AVROS x SP	0/0	*0/0*	Excluded	T
C2579	AVROS x SP	T/T	*0/0*	Excluded	D
C2599	AVROS x SP	0/0	*0/0*	Excluded	T
C2505	AVROS x SP	T/T	*T/T*	P	P
C2507	AVROS x SP	T/T	*T/T*	P	P
C2515	AVROS x SP	T/T	*T/T*	P	P
C2520	AVROS x SP	T/T	*T/T*	P	P
**C2537	AVROS x SP	T/T	*T/T*	P	P
C2538	AVROS x SP	T/T	*T/T*	P	P
C2576	AVROS x SP	T/T	*T/T*	P	P
C2577	AVROS x SP	T/T	*T/T*	P	P
C2578	AVROS x SP	T/T	*T/T*	P	P
C2582	AVROS x SP	T/T	*T/T*	P	P
C2586	AVROS x SP	T/T	*T/T*	P	P
C2587	AVROS x SP	T/T	*T/T*	P	P
C2591	AVROS x SP	T/T	*T/T*	P	P
C2595	AVROS x SP	T/T	*T/T*	P	P

C2647	AVROS x SP	T/T	*T/T*	P	P
C2648	AVROS x SP	T/T	*T/T*	P	P
**C2649	AVROS x SP	T/T	*T/T*	P	P
C2651	AVROS x SP	T/T	*T/T*	P	P
C2675	AVROS x SP	T/T	*T/T*	P	P
C2679	AVROS x SP	T/T	*T/T*	P	P
C2681	AVROS x SP	T/T	*T/T*	P	P
C2684	AVROS x SP	T/T	*T/T*	P	P
C2687	AVROS x SP	T/T	*T/T*	P	P
C2713	AVROS x SP	T/T	*T/T*	P	P
C2721	AVROS x SP	T/T	*T/T*	P	P
C2723	AVROS x SP	T/T	*T/T*	P	P
C2728	AVROS x SP	T/T	*T/T*	P	P
C2730	AVROS x SP	T/T	*T/T*	P	P
C2732	AVROS x SP	T/T	*T/T*	P	P
C2736	AVROS x SP	T/T	*T/T*	P	P
C2737	AVROS x SP	T/T	*T/T*	P	P
C2739	AVROS x SP	T/T	*T/T*	P	P
C2742	AVROS x SP	T/T	*T/T*	P	P
C2793	AVROS x SP	T/T	*T/T*	P	P
C2798	AVROS x SP	T/T	*T/T*	P	P
C2801	AVROS x SP	T/T	*T/T*	P	P
C2803	AVROS x SP	T/T	*T/T*	P	P
C2820	AVROS x SP	T/T	*T/T*	P	P
C2825	AVROS x SP	T/T	*T/T*	P	P
C2827	AVROS x SP	T/T	*T/T*	P	P
C2862	AVROS x SP	T/T	*T/T*	P	P
C2864	AVROS x SP	T/T	*T/T*	P	P
C2867	AVROS x SP	T/T	*T/T*	P	P
C2870	AVROS x SP	T/T	*T/T*	P	P
C2884	AVROS x SP	T/T	*T/T*	P	P
C2887	AVROS x SP	T/T	*T/T*	P	P
C2889	AVROS x SP	T/T	*T/T*	P	P
C2934	AVROS x SP	T/T	*T/T*	P	P
C2935	AVROS x SP	T/T	*T/T*	P	P
C2937	AVROS x SP	T/T	*T/T*	P	P
C2941	AVROS x SP	T/T	*T/T*	P	P
C2942	AVROS x SP	T/T	*T/T*	P	P
C2943	AVROS x SP	T/T	*T/T*	P	P
C2946	AVROS x SP	T/T	*T/T*	P	P
C2948	AVROS x SP	T/T	*T/T*	P	P
**C2967	AVROS x SP	T/T	*T/T*	P	P
**C2971	AVROS x SP	T/T	*T/T*	P	P

C2973	AVROS x SP	T/T	*T/T*	P	P
C2502	AVROS x SP	T/T	*T/A*	T	T
C2503	AVROS x SP	T/T	*T/A*	T	T
C2506	AVROS x SP	T/T	*T/A*	T	T
C2508	AVROS x SP	T/T	*T/A*	T	T
C2509	AVROS x SP	T/T	*T/A*	T	T
C2513	AVROS x SP	T/T	*T/A*	T	T
C2524	AVROS x SP	T/T	*T/A*	T	T
C2533	AVROS x SP	T/T	*T/A*	T	T
C2572	AVROS x SP	T/T	*T/A*	T	T
C2573	AVROS x SP	T/T	*T/A*	T	T
C2575	AVROS x SP	T/T	*T/A*	T	T
C2581	AVROS x SP	T/T	*T/A*	T	T
C2583	AVROS x SP	T/T	*T/A*	T	T
C2589	AVROS x SP	T/T	*T/A*	T	T
C2590	AVROS x SP	T/T	*T/A*	T	T
C2596	AVROS x SP	T/T	*T/A*	T	T
C2645	AVROS x SP	T/T	*T/A*	T	T
C2650	AVROS x SP	T/T	*T/A*	T	T
C2652	AVROS x SP	T/T	*T/A*	T	T
C2653	AVROS x SP	T/T	*T/A*	T	T
C2654	AVROS x SP	T/T	*T/A*	T	T
C2655	AVROS x SP	T/T	*T/A*	T	T
C2656	AVROS x SP	T/T	*T/A*	T	T
C2657	AVROS x SP	T/T	*T/A*	T	T
C2674	AVROS x SP	T/T	*T/A*	T	T
C2683	AVROS x SP	T/T	*T/A*	T	T
C2688	AVROS x SP	T/T	*T/A*	T	T
C2714	AVROS x SP	T/T	*T/A*	T	T
C2716	AVROS x SP	T/T	*T/A*	T	T
C2719	AVROS x SP	T/T	*T/A*	T	T
C2720	AVROS x SP	T/T	*T/A*	T	T
C2722	AVROS x SP	T/T	*T/A*	T	T
C2725	AVROS x SP	T/T	*T/A*	T	T
C2735	AVROS x SP	T/T	*T/A*	T	T
C2738	AVROS x SP	T/T	*T/A*	T	T
C2790	AVROS x SP	T/T	*T/A*	T	T
C2791	AVROS x SP	T/T	*T/A*	T	T
C2792	AVROS x SP	T/T	*T/A*	T	T
C2794	AVROS x SP	T/T	*T/A*	T	T
C2796	AVROS x SP	T/T	*T/A*	T	T
C2797	AVROS x SP	T/T	*T/A*	T	T
C2799	AVROS x SP	T/T	*T/A*	T	T

C2800	AVROS x SP	T/T	*T/A*	T	T
C2802	AVROS x SP	T/T	*T/A*	T	T
C2861	AVROS x SP	T/T	*T/A*	T	T
C2863	AVROS x SP	T/T	*T/A*	T	T
C2866	AVROS x SP	T/T	*T/A*	T	T
C2871	AVROS x SP	T/T	*T/A*	T	T
C2873	AVROS x SP	T/T	*T/A*	T	T
C2875	AVROS x SP	T/T	*T/A*	T	T
C2877	AVROS x SP	T/T	*T/A*	T	T
C2879	AVROS x SP	T/T	*T/A*	T	T
C2881	AVROS x SP	T/T	*T/A*	T	T
C2882	AVROS x SP	T/T	*T/A*	T	T
C2888	AVROS x SP	T/T	*T/A*	T	T
C2936	AVROS x SP	T/T	*T/A*	T	T
C2938	AVROS x SP	T/T	*T/A*	T	T
C2939	AVROS x SP	T/T	*T/A*	T	T
C2940	AVROS x SP	T/T	*T/A*	T	T
C2944	AVROS x SP	T/T	*T/A*	T	T
C2945	AVROS x SP	T/T	*T/A*	T	T
C2947	AVROS x SP	T/T	*T/A*	T	T
C2961	AVROS x SP	T/T	*T/A*	T	T
C2968	AVROS x SP	T/T	*T/A*	T	T
C2970	AVROS x SP	T/T	*T/A*	T	T
C2972	AVROS x SP	T/T	*T/A*	T	T
C2974	AVROS x SP	T/T	*T/A*	T	T
C2473	AVROS-SP x Ekona	T/T	*A/A*	D	D
C2474	AVROS-SP x Ekona	T/T	*A/A*	D	D
C2484	AVROS-SP x Ekona	T/T	*A/A*	D	D
C2618	AVROS-SP x Ekona	T/T	*A/A*	D	D
C2621	AVROS-SP x Ekona	T/T	*A/A*	D	D
C2622	AVROS-SP x Ekona	T/T	*A/A*	D	D
C2623	AVROS-SP x Ekona	T/T	*A/A*	D	D
C2626	AVROS-SP x Ekona	T/T	*A/A*	D	D
C2759	AVROS-SP x Ekona	T/T	*A/A*	D	D
C2763	AVROS-SP x Ekona	T/T	*A/A*	D	D
C2767	AVROS-SP x Ekona	T/T	*A/A*	D	D
C2769	AVROS-SP x Ekona	T/T	*A/A*	D	D
C2770	AVROS-SP x Ekona	T/T	*A/A*	D	D
C2771	AVROS-SP x Ekona	T/T	*A/A*	D	D
C2772	AVROS-SP x Ekona	T/T	*A/A*	D	D
C2907	AVROS-SP x Ekona	T/T	*A/A*	D	D
C2908	AVROS-SP x Ekona	T/T	*A/A*	D	D
C2912	AVROS-SP x Ekona	T/T	*A/A*	D	D

C2913	AVROS-SP x Ekona	T/T	*A/A*	D	D	
C2916	AVROS-SP x Ekona	T/T	*A/A*	D	D	
C2917	AVROS-SP x Ekona	T/T	*A/A*	D	D	
C2918	AVROS-SP x Ekona	T/T	*A/A*	D	D	
C2919	AVROS-SP x Ekona	T/T	*A/A*	D	D	
C2761	AVROS-SP x Ekona	0/0	*0/0*	Excluded	T	
C2765	AVROS-SP x Ekona	0/0	*0/0*	Excluded	T	
C2766	AVROS-SP x Ekona	T/T	*0/0*	Excluded	T	
C2472	AVROS-SP x Ekona	T/T	*T/A*	T	T	
C2475	AVROS-SP x Ekona	T/T	*T/A*	T	T	
C2476	AVROS-SP x Ekona	C/T	*T/A*	T	P	Miss
C2477	AVROS-SP x Ekona	C/T	*A/A*	T	T	
C2478	AVROS-SP x Ekona	T/T	*T/A*	T	T	
C2479	AVROS-SP x Ekona	C/T	*T/A*	T	P	Miss
C2480	AVROS-SP x Ekona	C/T	*T/A*	T	P	Miss
C2481	AVROS-SP x Ekona	T/T	*T/A*	T	T	
C2482	AVROS-SP x Ekona	C/T	*A/A*	T	T	
C2483	AVROS-SP x Ekona	T/T	*T/A*	T	T	
C2485	AVROS-SP x Ekona	C/T	*T/A*	T	P	Miss
C2615	AVROS-SP x Ekona	T/T	*T/A*	T	T	
C2616	AVROS-SP x Ekona	C/T	*T/A*	T	P	Miss
C2617	AVROS-SP x Ekona	C/T	*T/A*	T	P	Miss
C2619	AVROS-SP x Ekona	T/T	*T/A*	T	T	
C2620	AVROS-SP x Ekona	T/T	*T/A*	T	T	
C2624	AVROS-SP x Ekona	C/T	*T/A*	T	P	Miss
C2625	AVROS-SP x Ekona	C/T	*A/A*	T	T	
C2628	AVROS-SP x Ekona	T/T	*T/A*	T	T	
C2629	AVROS-SP x Ekona	T/T	*T/A*	T	T	
C2760	AVROS-SP x Ekona	T/T	*T/A*	T	T	
C2762	AVROS-SP x Ekona	C/T	*A/A*	T	T	
C2764	AVROS-SP x Ekona	C/T	*A/A*	T	T	
C2768	AVROS-SP x Ekona	C/T	*T/A*	T	P	Miss
C2773	AVROS-SP x Ekona	T/T	*T/A*	T	T	
C2774	AVROS-SP x Ekona	C/T	*A/A*	T	T	
C2905	AVROS-SP x Ekona	T/T	*T/A*	T	T	
C2906	AVROS-SP x Ekona	C/T	*A/A*	T	T	
C2909	AVROS-SP x Ekona	T/T	*T/A*	T	T	
C2911	AVROS-SP x Ekona	C/T	*T/A*	T	P	Miss
C2914	AVROS-SP x Ekona	C/T	*T/A*	T	P	Miss
C2558	Ekona	T/T	*A/A*	D	D	
C2561	Ekona	T/T	*A/A*	D	D	
C2562	Ekona	T/T	*A/A*	D	D	
C2563	Ekona	T/T	*A/A*	D	D	

C2566	Ekona	T/T	*A/A*	D	D
C2567	Ekona	T/T	*A/A*	D	D
C2569	Ekona	T/T	*A/A*	D	D
C2708	Ekona	T/T	*A/A*	D	D
C2709	Ekona	T/T	*A/A*	D	D
C2849	Ekona	T/T	*A/A*	D	D
C2850	Ekona	T/T	*A/A*	D	D
C2851	Ekona	T/T	*A/A*	D	D
C2848	Ekona	0/0	*0/0*	Excluded	T
C2564	Ekona	CC	*A/A*	P	P
C2565	Ekona	CC	*A/A*	P	P
C2701	Ekona	CC	*A/A*	P	P
C2703	Ekona	CC	*A/A*	P	P
C2707	Ekona	CC	*A/A*	P	P
C2710	Ekona	CC	*A/A*	P	P
C2711	Ekona	CC	*A/A*	P	P
C2855	Ekona	CC	*A/A*	P	P
C2858	Ekona	CC	*A/A*	P	P
C2860	Ekona	CC	*A/A*	P	P
C2557	Ekona	C/T	*A/A*	T	T
C2559	Ekona	C/T	*A/A*	T	T
C2560	Ekona	C/T	*A/A*	T	T
C2568	Ekona	C/T	*A/A*	T	T
C2704	Ekona	C/T	*A/A*	T	T
C2705	Ekona	C/T	*A/A*	T	T
C2706	Ekona	C/T	*A/A*	T	T
C2852	Ekona	C/T	*A/A*	T	T
C2853	Ekona	C/T	*A/A*	T	T
C2854	Ekona	C/T	*A/A*	T	T
C2856	Ekona	C/T	*A/A*	T	T
C2857	Ekona	C/T	*A/A*	T	T
C2859	Ekona	C/T	*A/A*	T	T

**Fertile *pisifera*. Excluded – Samples with missing data. D – *dura*; T – *tenera*; P – *pisifera*. For (M1, M2), both *T/T* and *A/A* are $sh^{Deli\ Dura}/sh^{Deli\ Dura}$ as wild type; M1 - *T/C* and *C/C* are $sh^{Deli\ Dura}/sh^{MPOB}$ and sh^{MPOB}/sh^{MPOB}; M2 - *T/A* and *T/T* are $sh^{Deli\ Dura}/sh^{AVROS}$ and sh^{AVROS}/sh^{AVROS}.

Supplementary Table 2. The genotype-phenotype analysis of fruit form in the second validation set based on haploinsufficiency model and genotypes of assayed samples.

Sample ID	Accession	Sh^{MPOB}	Sh^{AVROS}	Predicted Fruit Form	Observed Fruit Form	Accuracy
C2	AVROS	*T/T*	*A/A*	D	T	Miss
C2269	AVROS	*T/T*	*A/A*	D	D	
C2275	AVROS	*T/T*	*A/A*	D	D	
C2346	AVROS	*T/T*	*T/T*	P	P	
C2352	AVROS	*T/T*	*T/T*	P	P	
C2356	AVROS	*T/T*	*T/T*	P	P	
C1	AVROS	*T/T*	*T/A*	T	P	Miss
C3	AVROS	*T/T*	*T/A*	T	P	Miss
C2434	AVROS	*T/T*	*T/A*	T	T	
C2437	AVROS	*T/T*	*T/A*	T	T	
C2440	AVROS	*T/T*	*T/A*	T	T	
V920	Cameroon x Congo	*C/T*	*T/A*	P	P	
V927	Cameroon x Congo	*T//T*	*A/A*	D	T	Miss
V907	Cameroon x Congo	*T/T*	*A/A*	D	D	
V917	Cameroon x Congo	*T//T*	*A/A*	D	D	
V919	Cameroon x Congo	*T/T*	*A/A*	D	D	
V929	Cameroon x Congo	*T/T*	*A/A*	D	D	
V935	Cameroon x Congo	*T/T*	*A/A*	D	D	
V604	Cameroon x Congo	*T/T*	*A/A*	D	D	
V605	Cameroon x Congo	*T/T*	*A/A*	D	D	
V613	Cameroon x Congo	*T/T*	*A/A*	D	D	
V618	Cameroon x Congo	*T/T*	*A/A*	D	D	
V619	Cameroon x Congo	*T/T*	*A/A*	D	D	
V624	Cameroon x Congo	*T/T*	*A/A*	D	D	
V628	Cameroon x Congo	*T/T*	*A/A*	D	D	
V918	Cameroon x Congo	*C/T*	*T/A*	P	P	
V904	Cameroon x Congo	*C/T*	*T/A*	P	P	
V910	Cameroon x Congo	*C/T*	*T/A*	P	P	
V915	Cameroon x Congo	*C/T*	*T/A*	P	P	
V922	Cameroon x Congo	*C/T*	*T/A*	P	P	
V932	Cameroon x Congo	*C/T*	*T/A*	P	P	
V600	Cameroon x Congo	*C/C*	*A/A*	P	P	
V614	Cameroon x Congo	*C/C*	*A/A*	P	P	
V615	Cameroon x Congo	*C/C*	*A/A*	P	P	
V617	Cameroon x Congo	*C/C*	*A/A*	P	P	
V621	Cameroon x Congo	*C/C*	*A/A*	P	P	
V623	Cameroon x Congo	*C/C*	*A/A*	P	P	
V633	Cameroon x Congo	*C/C*	*A/A*	P	P	
V625	Cameroon x Congo	*T/T*	*T/T*	P	P	

V902	Cameroon x Congo	*C/T*	*A/A*	T	T	
V909	Cameroon x Congo	*C/T*	*A/A*	T	T	
V911	Cameroon x Congo	*C/T*	*A/A*	T	T	
V924	Cameroon x Congo	*C/T*	*A/A*	T	T	
V925	Cameroon x Congo	*C/T*	*A/A*	T	T	
V926	Cameroon x Congo	*C/T*	*A/A*	T	T	
V928	Cameroon x Congo	*C/T*	*A/A*	T	T	
V931	Cameroon x Congo	*C/T*	*A/A*	T	T	
V903	Cameroon x Congo	*T/T*	*T/A*	T	T	
V906	Cameroon x Congo	*T/T*	*T/A*	T	T	
V908	Cameroon x Congo	*T/T*	*T/A*	T	T	
V914	Cameroon x Congo	*T/T*	*T/A*	T	T	
V934	Cameroon x Congo	*T/T*	*T/A*	T	T	
V609	Cameroon x Congo	*C/T*	*A/A*	T	T	
V599	Cameroon x Congo	*C/T*	*A/A*	T	P	Miss
V601	Cameroon x Congo	*C/T*	*A/A*	T	T	
V607	Cameroon x Congo	*C/T*	*A/A*	T	T	
V608	Cameroon x Congo	*C/T*	*A/A*	T	T	
V610	Cameroon x Congo	*C/T*	*A/A*	T	T	
V611	Cameroon x Congo	*C/T*	*A/A*	T	T	
V612	Cameroon x Congo	*C/T*	*A/A*	T	T	
V616	Cameroon x Congo	*C/T*	*A/A*	T	T	
V622	Cameroon x Congo	*C/T*	*A/A*	T	T	
V627	Cameroon x Congo	*C/T*	*A/A*	T	T	
V629	Cameroon x Congo	*C/T*	*A/A*	T	T	
V630	Cameroon x Congo	*C/T*	*A/A*	T	T	
V631	Cameroon x Congo	*C/T*	*A/A*	T	T	
V632	Cameroon x Congo	*C/T*	*A/A*	T	T	
V626	Cameroon x Congo	*C/T*	*A/A*	T	T	
V643	Congo	*T/T*	*A/A*	D	D	
V674	Congo	*T/T*	*A/A*	D	D	
V637	Congo	*T/T*	*A/A*	D	D	
V648	Congo	*T/T*	*A/A*	D	D	
V665	Congo	*T/T*	*A/A*	D	D	
V668	Congo	*T/T*	*A/A*	D	D	
V670	Congo	*T/T*	*A/A*	D	D	
V672	Congo	*T/T*	*A/A*	D	D	
V675	Congo	*T/T*	*A/A*	D	D	
V679	Congo	*T/T*	*A/A*	D	D	
V688	Congo	*T/T*	*A/A*	D	D	
V694	Congo	*T/T*	*A/A*	D	D	
V699	Congo	*T/T*	*A/A*	D	D	
V639	Congo	*T/T*	*T/T*	P	P	

V671	Congo	*T/T*	*T/T*	P	P
V685	Congo	*T/T*	*T/T*	P	P
V686	Congo	*T/T*	*T/T*	P	P
V692	Congo	*T/T*	*T/T*	P	P
V697	Congo	*T/T*	*T/T*	P	P
V662	Congo	*T/T*	*A/A*	D	D
V695	Congo	*T/T*	*T/A*	T	T
V634	Congo	*T/T*	*T/A*	T	T
V635	Congo	*T/T*	*T/A*	T	T
V636	Congo	*T/T*	*T/A*	T	T
V638	Congo	*T/T*	*T/A*	T	T
V640	Congo	*T/T*	*T/A*	T	T
V641	Congo	*T/T*	*T/A*	T	T
V642	Congo	*T/T*	*T/A*	T	T
V646	Congo	*T/T*	*T/A*	T	T
V656	Congo	*T/T*	*T/A*	T	T
V658	Congo	*T/T*	*T/A*	T	T
V663	Congo	*T/T*	*T/A*	T	T
V667	Congo	*T/T*	*T/A*	T	T
V676	Congo	*T/T*	*T/A*	T	T
V682	Congo	*T/T*	*T/A*	T	T
V683	Congo	*T/T*	*T/A*	T	T
V684	Congo	*T/T*	*T/A*	T	T
V687	Congo	*T/T*	*T/A*	T	T
V693	Congo	*T/T*	*T/A*	T	T
V677	Congo	*T/T*	*T/A*	T	T
CA1	Deli-Nig x URT-Calabar	*T/T*	*A/A*	D	D
CA2	Deli-Nig x URT-Calabar	*T/T*	*A/A*	D	D
CA3	Deli-Nig x URT-Calabar	*T/T*	*A/A*	D	D
CA4	Deli-Nig x URT-Calabar	*T/T*	*A/A*	D	D
CA5	Deli-Nig x URT-Calabar	*T/T*	*A/A*	D	D
CA6	Deli-Nig x URT-Calabar	*T/T*	*A/A*	D	D
CA7	Deli-Nig x URT-Calabar	*T/T*	*A/A*	D	D
CA8	Deli-Nig x URT-Calabar	*T/T*	*A/A*	D	D
CA9	Deli-Nig x URT-Calabar	*T/T*	*A/A*	D	D
CA12	Deli-Nig x URT-Calabar	*T/T*	*A/A*	D	D
CA13	Deli-Nig x URT-Calabar	*T/T*	*A/A*	D	D
CAP142	Deli-Nig x URT-Calabar	*C/T*	*A/A*	T	T
CAP145	Deli-Nig x URT-Calabar	*C/T*	*A/A*	T	T
CAP148	Deli-Nig x URT-Calabar	*C/T*	*A/A*	T	T
CAP151	Deli-Nig x URT-Calabar	*C/T*	*A/A*	T	T
CAP152	Deli-Nig x URT-Calabar	*C/T*	*A/A*	T	T
CAP153	Deli-Nig x URT-Calabar	*C/T*	*A/A*	T	T

CAP155	Deli-Nig x URT-Calabar	*C/T*	*A/A*	T	T
CAP156	Deli-Nig x URT-Calabar	*C/T*	*A/A*	T	T
CAP157	Deli-Nig x URT-Calabar	*C/T*	*A/A*	T	T
CAP160	Deli-Nig x URT-Calabar	*C/T*	*A/A*	T	T
CAP161	Deli-Nig x URT-Calabar	*C/T*	*A/A*	T	T
CAP162	Deli-Nig x URT-Calabar	*C/T*	*A/A*	T	T
CAP164	Deli-Nig x URT-Calabar	*C/T*	*A/A*	T	T
CAP141	Deli-Nig x URT-Calabar	*T/T*	*T/A*	T	T
CAP143	Deli-Nig x URT-Calabar	*T/T*	*T/A*	T	T
CAP144	Deli-Nig x URT-Calabar	*T/T*	*T/A*	T	T
CAP146	Deli-Nig x URT-Calabar	*T/T*	*T/A*	T	T
CAP147	Deli-Nig x URT-Calabar	*T/T*	*T/A*	T	T
CAP149	Deli-Nig x URT-Calabar	*T/T*	*T/A*	T	T
CAP154	Deli-Nig x URT-Calabar	*T/T*	*T/A*	T	T
CAP158	Deli-Nig x URT-Calabar	*T/T*	*T/A*	T	T
CAP159	Deli-Nig x URT-Calabar	*T/T*	*T/A*	T	T
CAP163	Deli-Nig x URT-Calabar	*T/T*	*T/A*	T	T
CAP170	Deli-Nig x URT-Calabar	*C/T*	*A/A*	T	T
CAP172	Deli-Nig x URT-Calabar	*C/T*	*A/A*	T	T
CAP174	Deli-Nig x URT-Calabar	*C/T*	*A/A*	T	T
CAP175	Deli-Nig x URT-Calabar	*C/T*	*A/A*	T	T
CAP176	Deli-Nig x URT-Calabar	*C/T*	*A/A*	T	T
CAP177	Deli-Nig x URT-Calabar	*C/T*	*A/A*	T	T
CAP178	Deli-Nig x URT-Calabar	*C/T*	*A/A*	T	T
CAP180	Deli-Nig x URT-Calabar	*C/T*	*A/A*	T	T
CAP181	Deli-Nig x URT-Calabar	*C/T*	*A/A*	T	T
CAP182	Deli-Nig x URT-Calabar	*C/T*	*A/A*	T	T
CAP187	Deli-Nig x URT-Calabar	*C/T*	*A/A*	T	T
CAP188	Deli-Nig x URT-Calabar	*C/T*	*A/A*	T	T
CAP189	Deli-Nig x URT-Calabar	*C/T*	*A/A*	T	T
CAP165	Deli-Nig x URT-Calabar	*T/T*	*T/A*	T	T
CAP166	Deli-Nig x URT-Calabar	*T/T*	*T/A*	T	T
CAP167	Deli-Nig x URT-Calabar	*T/T*	*T/A*	T	T
CAP169	Deli-Nig x URT-Calabar	*T/T*	*T/A*	T	T
CAP173	Deli-Nig x URT-Calabar	*T/T*	*T/A*	T	T
CAP179	Deli-Nig x URT-Calabar	*T/T*	*T/A*	T	T
CAP184	Deli-Nig x URT-Calabar	*T/T*	*T/A*	T	T
CAP185	Deli-Nig x URT-Calabar	*T/T*	*T/A*	T	T
CAP186	Deli-Nig x URT-Calabar	*T/T*	*T/A*	T	T
CAP190	Deli-Nig x URT-Calabar	*T/T*	*T/A*	T	T
CAP191	Deli-Nig x URT-Calabar	*T/T*	*T/A*	T	T
CAP192	Deli-Nig x URT-Calabar	*T/T*	*T/A*	T	T
CAP219	Deli-Nig x URT-Calabar	*C/T*	*A/A*	T	T

CAP194	Deli-Nig x URT-Calabar	*C/T*	*A/A*	T	T
CAP197	Deli-Nig x URT-Calabar	*C/T*	*A/A*	T	T
CAP198	Deli-Nig x URT-Calabar	*C/T*	*A/A*	T	T
CAP199	Deli-Nig x URT-Calabar	*C/T*	*A/A*	T	T
CAP200	Deli-Nig x URT-Calabar	*C/T*	*A/A*	T	T
CAP201	Deli-Nig x URT-Calabar	*C/T*	*A/A*	T	T
CAP202	Deli-Nig x URT-Calabar	*C/T*	*A/A*	T	T
CAP203	Deli-Nig x URT-Calabar	*C/T*	*A/A*	T	T
CAP204	Deli-Nig x URT-Calabar	*C/T*	*A/A*	T	T
CAP205	Deli-Nig x URT-Calabar	*C/T*	*A/A*	T	T
CAP207	Deli-Nig x URT-Calabar	*C/T*	*A/A*	T	T
CAP209	Deli-Nig x URT-Calabar	*C/T*	*A/A*	T	T
CAP212	Deli-Nig x URT-Calabar	*C/T*	*A/A*	T	T
CAP215	Deli-Nig x URT-Calabar	*C/T*	*A/A*	T	T
CAP216	Deli-Nig x URT-Calabar	*C/T*	*A/A*	T	T
CAP217	Deli-Nig x URT-Calabar	*C/T*	*A/A*	T	T
CAP220	Deli-Nig x URT-Calabar	*C/T*	*A/A*	T	T
CAP193	Deli-Nig x URT-Calabar	*T/T*	*T/A*	T	T
CAP195	Deli-Nig x URT-Calabar	*T/T*	*T/A*	T	T
CAP196	Deli-Nig x URT-Calabar	*T/T*	*T/A*	T	T
CAP208	Deli-Nig x URT-Calabar	*T/T*	*T/A*	T	T
CAP210	Deli-Nig x URT-Calabar	*T/T*	*T/A*	T	T
CAP211	Deli-Nig x URT-Calabar	*T/T*	*T/A*	T	T
CAP213	Deli-Nig x URT-Calabar	*T/T*	*T/A*	T	T
CAP214	Deli-Nig x URT-Calabar	*T/T*	*T/A*	T	T
CAP218	Deli-Nig x URT-Calabar	*T/T*	*T/A*	T	T
CAP221	Deli-Nig x URT-Calabar	*C/T*	*A/A*	T	T
CAP224	Deli-Nig x URT-Calabar	*C/T*	*A/A*	T	T
CAP225	Deli-Nig x URT-Calabar	*C/T*	*A/A*	T	T
CAP226	Deli-Nig x URT-Calabar	*C/T*	*A/A*	T	T
CAP229	Deli-Nig x URT-Calabar	*C/T*	*A/A*	T	T
CAP232	Deli-Nig x URT-Calabar	*C/T*	*A/A*	T	T
CAP222	Deli-Nig x URT-Calabar	*T/T*	*T/A*	T	T
CAP227	Deli-Nig x URT-Calabar	*T/T*	*T/A*	T	T
CAP231	Deli-Nig x URT-Calabar	*T/T*	*T/A*	T	T
V700	MPOB	*T/T*	*A/A*	D	D
V701	MPOB	*T/T*	*A/A*	D	D
V702	MPOB	*T/T*	*A/A*	D	D
V703	MPOB	*T/T*	*A/A*	D	D
V704	MPOB	*T/T*	*A/A*	D	D
V705	MPOB	*T/T*	*A/A*	D	D
V706	MPOB	*T/T*	*A/A*	D	D
V707	MPOB	*T/T*	*A/A*	D	D

V708	MPOB	*T/T*	*A/A*	D	D
V710	MPOB	*T/T*	*A/A*	D	D
V711	MPOB	*T/T*	*A/A*	D	D
V712	MPOB	*T/T*	*A/A*	D	D
V713	MPOB	*T/T*	*A/A*	D	D
V714	MPOB	*T/T*	*A/A*	D	D
V715	MPOB	*T/T*	*A/A*	D	D
V716	MPOB	*T/T*	*A/A*	D	D
V717	MPOB	*T/T*	*A/A*	D	D
V718	MPOB	*T/T*	*A/A*	D	D
V719	MPOB	*T/T*	*A/A*	D	D
V720	MPOB	*T/T*	*A/A*	D	D
V721	MPOB	*T/T*	*A/A*	D	D
V722	MPOB	*T/T*	*A/A*	D	D
V723	MPOB	*T/T*	*A/A*	D	D
V724	MPOB	*T/T*	*A/A*	D	D
V725	MPOB	*T/T*	*A/A*	D	D
V726	MPOB	*T/T*	*A/A*	D	D
V727	MPOB	*T/T*	*A/A*	D	D
V728	MPOB	*T/T*	*A/A*	D	D
V729	MPOB	*T/T*	*A/A*	D	D
V730	MPOB	*T/T*	*A/A*	D	D
V731	MPOB	*T/T*	*A/A*	D	D
V732	MPOB	*T/T*	*A/A*	D	D
V733	MPOB	*T/T*	*A/A*	D	D
V734	MPOB	*T/T*	*A/A*	D	D
V735	MPOB	*T/T*	*A/A*	D	D
V736	MPOB	*T/T*	*A/A*	D	D
V737	MPOB	*T/T*	*A/A*	D	D
V738	MPOB	*T/T*	*A/A*	D	D
V739	MPOB	*T/T*	*A/A*	D	D
V740	MPOB	*T/T*	*A/A*	D	D
V741	MPOB	*T/T*	*A/A*	D	D
V742	MPOB	*T/T*	*A/A*	D	D
V744	MPOB	*T/T*	*A/A*	D	D
V745	MPOB	*T/T*	*A/A*	D	D
V746	MPOB	*T/T*	*A/A*	D	D
V747	MPOB	*T/T*	*A/A*	D	D
V748	MPOB	*T/T*	*A/A*	D	D
V749	MPOB	*T/T*	*A/A*	D	D
V750	MPOB	*T/T*	*A/A*	D	D
V752	MPOB	*T/T*	*A/A*	D	D
V753	MPOB	*T/T*	*A/A*	D	D

V754	MPOB	*T/T*	*A/A*	D	D
V755	MPOB	*T/T*	*A/A*	D	D
V756	MPOB	*T/T*	*A/A*	D	D
V757	MPOB	*T/T*	*A/A*	D	D
V758	MPOB	*T/T*	*A/A*	D	D
V760	MPOB	*T/T*	*A/A*	D	D
V763	MPOB	*T/T*	*A/A*	D	D
V764	MPOB	*T/T*	*A/A*	D	D
V765	MPOB	*T/T*	*A/A*	D	D
V766	MPOB	*T/T*	*A/A*	D	D
V768	MPOB	*T/T*	*A/A*	D	D
V769	MPOB	*T/T*	*A/A*	D	D
V770	MPOB	*T/T*	*A/A*	D	D
V771	MPOB	*T/T*	*A/A*	D	D
V773	MPOB	*T/T*	*A/A*	D	D
V774	MPOB	*T/T*	*A/A*	D	D
V775	MPOB	*T/T*	*A/A*	D	D
V776	MPOB	*T/T*	*A/A*	D	D
V777	MPOB	*T/T*	*A/A*	D	D
V778	MPOB	*T/T*	*A/A*	D	D
V780	MPOB	*T/T*	*A/A*	D	D
V782	MPOB	*T/T*	*A/A*	D	D
V784	MPOB	*T/T*	*A/A*	D	D
V785	MPOB	*T/T*	*A/A*	D	D
V786	MPOB	*T/T*	*A/A*	D	D
V787	MPOB	*T/T*	*A/A*	D	D
V788	MPOB	*T/T*	*A/A*	D	D
V789	MPOB	*T/T*	*A/A*	D	D
V790	MPOB	*T/T*	*A/A*	D	D
V791	MPOB	*T/T*	*A/A*	D	D
V792	MPOB	*T/T*	*A/A*	D	D
V793	MPOB	*T/T*	*A/A*	D	D
V794	MPOB	*T/T*	*A/A*	D	D
V762	MPOB	*T/T*	*A/A*	D	D
V779	MPOB	*T/T*	*A/A*	D	D
V795	MPOB	*T/T*	*A/A*	D	D
V796	MPOB	*T/T*	*A/A*	D	D
V797	MPOB	*T/T*	*A/A*	D	D
V798	MPOB	*T/T*	*A/A*	D	D
V799	MPOB	*T/T*	*A/A*	D	D
V800	MPOB	*T/T*	*A/A*	D	D
V801	MPOB	*T/T*	*A/A*	D	D
V802	MPOB	*T/T*	*A/A*	D	D

V803	MPOB	*T/T*	*A/A*	D	D
V804	MPOB	*T/T*	*A/A*	D	D
V805	MPOB	*T/T*	*A/A*	D	D
V806	MPOB	*T/T*	*A/A*	D	D
V807	MPOB	*T/T*	*A/A*	D	D
V808	MPOB	*T/T*	*A/A*	D	D
V809	MPOB	*T/T*	*A/A*	D	D
V810	MPOB	*T/T*	*A/A*	D	D
V811	MPOB	*T/T*	*A/A*	D	D
V812	MPOB	*T/T*	*A/A*	D	D
V813	MPOB	*T/T*	*A/A*	D	D
V816	MPOB	*T/T*	*A/A*	D	D
V817	MPOB	*T/T*	*A/A*	D	D
V819	MPOB	*T/T*	*A/A*	D	D
V820	MPOB	*T/T*	*A/A*	D	D
V821	MPOB	*T/T*	*A/A*	D	D
V822	MPOB	*T/T*	*A/A*	D	D
V823	MPOB	*T/T*	*A/A*	D	D
V824	MPOB	*T/T*	*A/A*	D	D
V825	MPOB	*T/T*	*A/A*	D	D
V826	MPOB	*T/T*	*A/A*	D	D
V827	MPOB	*T/T*	*A/A*	D	D
V828	MPOB	*T/T*	*A/A*	D	D
V829	MPOB	*T/T*	*A/A*	D	D
V830	MPOB	*T/T*	*A/A*	D	D
V831	MPOB	*T/T*	*A/A*	D	D
V832	MPOB	*T/T*	*A/A*	D	D
V833	MPOB	*T/T*	*A/A*	D	D
V834	MPOB	*T/T*	*A/A*	D	D
V835	MPOB	*T/T*	*A/A*	D	D
V836	MPOB	*T/T*	*A/A*	D	D
V837	MPOB	*T/T*	*A/A*	D	D
V838	MPOB	*T/T*	*A/A*	D	D
V840	MPOB	*T/T*	*A/A*	D	D
V841	MPOB	*T/T*	*A/A*	D	D
V842	MPOB	*T/T*	*A/A*	D	D
V843	MPOB	*T/T*	*A/A*	D	D
V844	MPOB	*T/T*	*A/A*	D	D
V845	MPOB	*T/T*	*A/A*	D	D
V846	MPOB	*T/T*	*A/A*	D	D
V847	MPOB	*T/T*	*A/A*	D	D
V848	MPOB	*T/T*	*A/A*	D	D
V004	Nigerian x AVROS	*T/T*	*A/A*	D	D

V127	Nigerian x AVROS	*T/T*	*A/A*	D	D
V097	Nigerian x AVROS	*T/T*	*A/A*	D	D
V098	Nigerian x AVROS	*T/T*	*A/A*	D	D
V126	Nigerian x AVROS	*T/T*	*A/A*	D	D
V129	Nigerian x AVROS	*T/T*	*A/A*	D	D
V102	Nigerian x AVROS	*T/T*	*A/A*	D	D
V148	Nigerian x AVROS	*T/T*	*A/A*	D	D
V141	Nigerian x AVROS	*T/T*	*A/A*	D	D
V105	Nigerian x AVROS	*T/T*	*A/A*	D	D
V107	Nigerian x AVROS	*T/T*	*A/A*	D	D
V132	Nigerian x AVROS	*T/T*	*A/A*	D	D
V109	Nigerian x AVROS	*T/T*	*A/A*	D	D
V149	Nigerian x AVROS	*T/T*	*A/A*	D	D
V123	Nigerian x AVROS	*T/T*	*A/A*	D	D
V137	Nigerian x AVROS	*T/T*	*A/A*	D	D
V113	Nigerian x AVROS	*T/T*	*A/A*	D	D
V116	Nigerian x AVROS	*T/T*	*A/A*	D	D
V142	Nigerian x AVROS	*T/T*	*A/A*	D	D
V119	Nigerian x AVROS	*T/T*	*A/A*	D	D
V136	Nigerian x AVROS	*T/T*	*A/A*	D	D
V124	Nigerian x AVROS	*T/T*	*A/A*	D	D
V055	Nigerian x AVROS	*T/T*	*A/A*	D	D
V029	Nigerian x AVROS	*T/T*	*A/A*	D	D
V018	Nigerian x AVROS	*T/T*	*A/A*	D	D
V012	Nigerian x AVROS	*T/T*	*A/A*	D	D
V017	Nigerian x AVROS	*T/T*	*A/A*	D	D
V053	Nigerian x AVROS	*T/T*	*A/A*	D	D
V069	Nigerian x AVROS	*T/T*	*A/A*	D	D
V094	Nigerian x AVROS	*T/T*	*A/A*	D	D
V040	Nigerian x AVROS	*T/T*	*A/A*	D	D
V071	Nigerian x AVROS	*T/T*	*A/A*	D	D
V075	Nigerian x AVROS	*T/T*	*A/A*	D	D
V006	Nigerian x AVROS	*T/T*	*A/A*	D	D
V087	Nigerian x AVROS	*T/T*	*A/A*	D	D
V034	Nigerian x AVROS	*T/T*	*A/A*	D	D
V054	Nigerian x AVROS	*T/T*	*A/A*	D	D
V043	Nigerian x AVROS	*T/T*	*A/A*	D	D
V060	Nigerian x AVROS	*T/T*	*A/A*	D	D
V027	Nigerian x AVROS	*T/T*	*A/A*	D	D
V041	Nigerian x AVROS	*T/T*	*A/A*	D	D
V068	Nigerian x AVROS	*T/T*	*A/A*	D	D
V063	Nigerian x AVROS	*T/T*	*A/A*	D	D
V067	Nigerian x AVROS	*T/T*	*A/A*	D	D

V050	Nigerian x AVROS	*T/T*	*A/A*	D	D
V024	Nigerian x AVROS	*T/T*	*A/A*	D	D
V074	Nigerian x AVROS	*T/T*	*A/A*	D	D
V026	Nigerian x AVROS	*T/T*	*A/A*	D	D
V016	Nigerian x AVROS	*T/T*	*A/A*	D	D
V022	Nigerian x AVROS	*T/T*	*A/A*	D	D
V037	Nigerian x AVROS	*T/T*	*A/A*	D	D
V044	Nigerian x AVROS	*T/T*	*A/A*	D	D
V010	Nigerian x AVROS	*T/T*	*A/A*	D	D
V073	Nigerian x AVROS	*T/T*	*A/A*	D	D
V038	Nigerian x AVROS	*T/T*	*A/A*	D	D
V002	Nigerian x AVROS	*T/T*	*A/A*	D	D
V070	Nigerian x AVROS	*T/T*	*A/A*	D	D
V052	Nigerian x AVROS	*T/T*	*A/A*	D	D
V059	Nigerian x AVROS	*T/T*	*A/A*	D	D
V090	Nigerian x AVROS	*T/T*	*A/A*	D	D
V056	Nigerian x AVROS	*T/T*	*A/A*	D	D
V057	Nigerian x AVROS	*T/T*	*A/A*	D	D
V031	Nigerian x AVROS	*T/T*	*A/A*	D	D
V051	Nigerian x AVROS	*T/T*	*A/A*	D	D
V061	Nigerian x AVROS	*T/T*	*A/A*	D	D
V021	Nigerian x AVROS	*T/T*	*A/A*	D	D
V091	Nigerian x AVROS	*T/T*	*A/A*	D	D
V020	Nigerian x AVROS	*T/T*	*A/A*	D	D
V092	Nigerian x AVROS	*T/T*	*A/A*	D	D
V134	Nigerian x AVROS	*C/T*	*T/A*	P	P
V108	Nigerian x AVROS	*C/T*	*T/A*	P	P
V147	Nigerian x AVROS	*C/T*	*T/A*	P	P
V121	Nigerian x AVROS	*C/T*	*T/A*	P	P
V099	Nigerian x AVROS	*T/T*	*T/T*	P	P
V064	Nigerian x AVROS	*C/C*	*A/A*	P	P
V072	Nigerian x AVROS	*C/T*	*A/A*	T	T
V035	Nigerian x AVROS	*C/T*	*A/A*	T	T
V065	Nigerian x AVROS	*C/T*	*A/A*	T	T
V011	Nigerian x AVROS	*C/T*	*A/A*	T	T
V049	Nigerian x AVROS	*C/T*	*A/A*	T	T
V036	Nigerian x AVROS	*C/T*	*A/A*	T	T
V086	Nigerian x AVROS	*C/T*	*A/A*	T	T
V001	Nigerian x AVROS	*C/T*	*A/A*	T	T
V033	Nigerian x AVROS	*C/T*	*A/A*	T	T
V079	Nigerian x AVROS	*C/T*	*A/A*	T	T
V008	Nigerian x AVROS	*C/T*	*A/A*	T	T
V039	Nigerian x AVROS	*C/T*	*A//A*	T	T

V078	Nigerian x AVROS	*C/T*	*A//A*	T	T
V062	Nigerian x AVROS	*C/T*	*A/A*	T	T
V095	Nigerian x AVROS	*C/T*	*A/A*	T	T
V096	Nigerian x AVROS	*C/T*	*A/A*	T	T
V143	Nigerian x AVROS	*C/T*	*A/A*	T	T
V100	Nigerian x AVROS	*C/T*	*A/A*	T	T
V130	Nigerian x AVROS	*C/T*	*A/A*	T	T
V144	Nigerian x AVROS	*C/T*	*A/A*	T	T
V133	Nigerian x AVROS	*C/T*	*A/A*	T	T
V101	Nigerian x AVROS	*C/T*	*A/A*	T	T
V128	Nigerian x AVROS	*C/T*	*A/A*	T	T
V110	Nigerian x AVROS	*C/T*	*A/A*	T	T
V112	Nigerian x AVROS	*C/T*	*A/A*	T	T
V125	Nigerian x AVROS	*C/T*	*A/A*	T	T
V114	Nigerian x AVROS	*C/T*	*A/A*	T	T
V117	Nigerian x AVROS	*C/T*	*A/A*	T	T
V146	Nigerian x AVROS	*C/T*	*A/A*	T	T
V120	Nigerian x AVROS	*C/T*	*A/A*	T	T
V140	Nigerian x AVROS	*C/T*	*A/A*	T	T
V131	Nigerian x AVROS	*T/T*	*T/A*	T	T
V103	Nigerian x AVROS	*T/T*	*T/A*	T	T
V104	Nigerian x AVROS	*T/T*	*T/A*	T	T
V139	Nigerian x AVROS	*T/T*	*T/A*	T	T
V122	Nigerian x AVROS	*T/T*	*T/A*	T	T
V106	Nigerian x AVROS	*T/T*	*T/A*	T	T
V135	Nigerian x AVROS	*T/T*	*T/A*	T	T
V111	Nigerian x AVROS	*T/T*	*T/A*	T	T
V138	Nigerian x AVROS	*T/T*	*T/A*	T	T
V115	Nigerian x AVROS	*T/T*	*T/A*	T	T
V145	Nigerian x AVROS	*T/T*	*T/A*	T	T
V118	Nigerian x AVROS	*T/T*	*T/A*	T	T
V005	Nigerian x AVROS	*C/T*	*A/A*	T	T
V093	Nigerian x AVROS	*C/T*	*A/A*	T	T
V066	Nigerian x AVROS	*C/T*	*A/A*	T	T
V081	Nigerian x AVROS	*C/T*	*A/A*	T	T
V028	Nigerian x AVROS	*C/T*	*A/A*	T	T
V013	Nigerian x AVROS	*C/T*	*A/A*	T	T
V015	Nigerian x AVROS	*C/T*	*A/A*	T	T
V047	Nigerian x AVROS	*C/T*	*A/A*	T	T
V007	Nigerian x AVROS	*C/T*	*A/A*	T	T
V083	Nigerian x AVROS	*C/T*	*A/A*	T	T
V032	Nigerian x AVROS	*C/T*	*A/A*	T	T
V082	Nigerian x AVROS	*C/T*	*A/A*	T	T

V019	Nigerian x AVROS	*C/T*	*A/A*	T	T
V025	Nigerian x AVROS	*C/T*	*A/A*	T	T
V084	Nigerian x AVROS	*C/T*	*A/A*	T	T
V042	Nigerian x AVROS	*C/T*	*A/A*	T	T
V048	Nigerian x AVROS	*C/T*	*A/A*	T	T
V076	Nigerian x AVROS	*C/T*	*A/A*	T	T
V089	Nigerian x AVROS	*C/T*	*A/A*	T	T
V088	Nigerian x AVROS	*C/T*	*A/A*	T	T
V023	Nigerian x AVROS	*C/T*	*A/A*	T	T
V085	Nigerian x AVROS	*C/T*	*A/A*	T	T
V046	Nigerian x AVROS	*C/T*	*A/A*	T	T
V080	Nigerian x AVROS	*C/T*	*A/A*	T	T
V009	Nigerian x AVROS	*C/T*	*A/A*	T	T
V077	Nigerian x AVROS	*C/T*	*A/A*	T	T
V003	Nigerian x AVROS	*C/T*	*A/A*	T	T
V030	Nigerian x AVROS	*C/T*	*A/A*	T	T
V045	Nigerian x AVROS	*C/T*	*A/A*	T	T
V363	Nigerian x AVROS	*T/T*	*T/A*	T	T
V364	Nigerian x AVROS	*T/T*	*T/A*	T	T
V365	Nigerian x AVROS	*T/T*	*T/A*	T	T
V366	Nigerian x AVROS	*T/T*	*T/A*	T	T
V370	Nigerian x AVROS	*T/T*	*T/A*	T	T
V371	Nigerian x AVROS	*T/T*	*T/A*	T	T
V372	Nigerian x AVROS	*T/T*	*T/A*	T	T
V376	Nigerian x AVROS	*T/T*	*T/A*	T	T
V378	Nigerian x AVROS	*T/T*	*T/A*	T	T
V379	Nigerian x AVROS	*T/T*	*T/A*	T	T
V380	Nigerian x AVROS	*T/T*	*T/A*	T	T
V382	Nigerian x AVROS	*T/T*	*T/A*	T	T
V383	Nigerian x AVROS	*T/T*	*T/A*	T	T
V384	Nigerian x AVROS	*T/T*	*T/A*	T	T
V388	Nigerian x AVROS	*T/T*	*T/A*	T	T
V389	Nigerian x AVROS	*T/T*	*T/A*	T	T
V390	Nigerian x AVROS	*T/T*	*T/A*	T	T
V391	Nigerian x AVROS	*T/T*	*T/A*	T	T
V392	Nigerian x AVROS	*T/T*	*T/A*	T	T
V393	Nigerian x AVROS	*T/T*	*T/A*	T	T
V394	Nigerian x AVROS	*T/T*	*T/A*	T	T
V395	Nigerian x AVROS	*T/T*	*T/A*	T	T
V396	Nigerian x AVROS	*T/T*	*T/A*	T	T
V397	Nigerian x AVROS	*T/T*	*T/A*	T	T
V398	Nigerian x AVROS	*T/T*	*T/A*	T	T
V399	Nigerian x AVROS	*T/T*	*T/A*	T	T

V400	Nigerian x AVROS	*T/T*	*T/A*	T	T
V401	Nigerian x AVROS	*T/T*	*T/A*	T	T
V404	Nigerian x AVROS	*T/T*	*T/A*	T	T
V405	Nigerian x AVROS	*T/T*	*T/A*	T	T
V406	Nigerian x AVROS	*T/T*	*T/A*	T	T
V407	Nigerian x AVROS	*T/T*	*T/A*	T	T
V408	Nigerian x AVROS	*T/T*	*T/A*	T	T
V409	Nigerian x AVROS	*T/T*	*T/A*	T	T
V410	Nigerian x AVROS	*T/T*	*T/A*	T	T
V411	Nigerian x AVROS	*T/T*	*T/A*	T	T
V413	Nigerian x AVROS	*T/T*	*T/A*	T	T
V414	Nigerian x AVROS	*T/T*	*T/A*	T	T
V415	Nigerian x AVROS	*T/T*	*T/A*	T	T
V416	Nigerian x AVROS	*T/T*	*T/A*	T	T
V417	Nigerian x AVROS	*T/T*	*T/A*	T	T
V418	Nigerian x AVROS	*T/T*	*T/A*	T	T
V419	Nigerian x AVROS	*T/T*	*T/A*	T	T
V420	Nigerian x AVROS	*T/T*	*T/A*	T	T
V422	Nigerian x AVROS	*T/T*	*T/A*	T	T
V423	Nigerian x AVROS	*T/T*	*T/A*	T	T
V424	Nigerian x AVROS	*T/T*	*T/A*	T	T
V425	Nigerian x AVROS	*T/T*	*T/A*	T	T
V426	Nigerian x AVROS	*T/T*	*T/A*	T	T
V427	Nigerian x AVROS	*T/T*	*T/A*	T	T
V428	Nigerian x AVROS	*T/T*	*T/A*	T	T
V429	Nigerian x AVROS	*T/T*	*T/A*	T	T
V430	Nigerian x AVROS	*T/T*	*T/A*	T	T
V431	Nigerian x AVROS	*T/T*	*T/A*	T	T
V432	Nigerian x AVROS	*T/T*	*T/A*	T	T
V433	Nigerian x AVROS	*T/T*	*T/A*	T	T
V434	Nigerian x AVROS	*T/T*	*T/A*	T	T
V435	Nigerian x AVROS	*T/T*	*T/A*	T	T
V436	Nigerian x AVROS	*T/T*	*T/A*	T	T
V437	Nigerian x AVROS	*T/T*	*T/A*	T	T
V438	Nigerian x AVROS	*T/T*	*T/A*	T	T
V439	Nigerian x AVROS	*T/T*	*T/A*	T	T
V440	Nigerian x AVROS	*T/T*	*T/A*	T	T
V441	Nigerian x AVROS	*T/T*	*T/A*	T	T
V442	Nigerian x AVROS	*T/T*	*T/A*	T	T
V443	Nigerian x AVROS	*T/T*	*T/A*	T	T
V444	Nigerian x AVROS	*T/T*	*T/A*	T	T
V445	Nigerian x AVROS	*T/T*	*T/A*	T	T
V446	Nigerian x AVROS	*T/T*	*T/A*	T	T

V447	Nigerian x AVROS	*T/T*	*T/A*	T	T
V448	Nigerian x AVROS	*T/T*	*T/A*	T	T
V449	Nigerian x AVROS	*T/T*	*T/A*	T	T
V450	Nigerian x AVROS	*T/T*	*T/A*	T	T
V451	Nigerian x AVROS	*T/T*	*T/A*	T	T
V452	Nigerian x AVROS	*T/T*	*T/A*	T	T
V454	Nigerian x AVROS	*T/T*	*T/A*	T	T
V456	Nigerian x AVROS	*T/T*	*T/A*	T	T
V457	Nigerian x AVROS	*T/T*	*T/A*	T	T
V459	Nigerian x AVROS	*T/T*	*T/A*	T	T
V460	Nigerian x AVROS	*T/T*	*T/A*	T	T
V461	Nigerian x AVROS	*T/T*	*T/A*	T	T
V462	Nigerian x AVROS	*T/T*	*T/A*	T	T
V463	Nigerian x AVROS	*T/T*	*T/A*	T	T
V464	Nigerian x AVROS	*T/T*	*T/A*	T	T
V465	Nigerian x AVROS	*T/T*	*T/A*	T	T
V466	Nigerian x AVROS	*T/T*	*T/A*	T	T
V467	Nigerian x AVROS	*T/T*	*T/A*	T	T
V469	Nigerian x AVROS	*T/T*	*T/A*	T	T
V470	Nigerian x AVROS	*T/T*	*T/A*	T	T
V471	Nigerian x AVROS	*T/T*	*T/A*	T	T
V472	Nigerian x AVROS	*T/T*	*T/A*	T	T
V473	Nigerian x AVROS	*T/T*	*T/A*	T	T
V474	Nigerian x AVROS	*T/T*	*T/A*	T	T
V475	Nigerian x AVROS	*T/T*	*T/A*	T	T
V476	Nigerian x AVROS	*T/T*	*T/A*	T	T
V477	Nigerian x AVROS	*T/T*	*T/A*	T	T
V478	Nigerian x AVROS	*T/T*	*T/A*	T	T
V479	Nigerian x AVROS	*T/T*	*T/A*	T	T
V480	Nigerian x AVROS	*T/T*	*T/A*	T	T
V482	Nigerian x AVROS	*T/T*	*T/A*	T	T
V484	Nigerian x AVROS	*T/T*	*T/A*	T	T
V485	Nigerian x AVROS	*T/T*	*T/A*	T	T
V486	Nigerian x AVROS	*T/T*	*T/A*	T	T
V487	Nigerian x AVROS	*T/T*	*T/A*	T	T
V488	Nigerian x AVROS	*T/T*	*T/A*	T	T
V489	Nigerian x AVROS	*T/T*	*T/A*	T	T
V490	Nigerian x AVROS	*T/T*	*T/A*	T	T
V492	Nigerian x AVROS	*T/T*	*T/A*	T	T
V493	Nigerian x AVROS	*T/T*	*T/A*	T	T
V494	Nigerian x AVROS	*T/T*	*T/A*	T	T
V495	Nigerian x AVROS	*T/T*	*T/A*	T	T
V496	Nigerian x AVROS	*T/T*	*T/A*	T	T

V499	Nigerian x AVROS	*T/T*	*T/A*	T	T
V500	Nigerian x AVROS	*T/T*	*T/A*	T	T
V502	Nigerian x AVROS	*T/T*	*T/A*	T	T
V503	Nigerian x AVROS	*T/T*	*T/A*	T	T
V504	Nigerian x AVROS	*T/T*	*T/A*	T	T
V505	Nigerian x AVROS	*T/T*	*T/A*	T	T
V506	Nigerian x AVROS	*T/T*	*T/A*	T	T
V507	Nigerian x AVROS	*T/T*	*T/A*	T	T
V508	Nigerian x AVROS	*T/T*	*T/A*	T	T
V509	Nigerian x AVROS	*T/T*	*T/A*	T	T
V510	Nigerian x AVROS	*T/T*	*T/A*	T	T
V511	Nigerian x AVROS	*T/T*	*T/A*	T	T
V512	Nigerian x AVROS	*T/T*	*T/A*	T	T
V513	Nigerian x AVROS	*T/T*	*T/A*	T	T
V514	Nigerian x AVROS	*T/T*	*T/A*	T	T
V515	Nigerian x AVROS	*T/T*	*T/A*	T	T
V516	Nigerian x AVROS	*T/T*	*T/A*	T	T
V517	Nigerian x AVROS	*T/T*	*T/A*	T	T
V518	Nigerian x AVROS	*T/T*	*T/A*	T	T
V520	Nigerian x AVROS	*T/T*	*T/A*	T	T
V521	Nigerian x AVROS	*T/T*	*T/A*	T	T
V522	Nigerian x AVROS	*T/T*	*T/A*	T	T
V150	Tanzanian	*T/T*	*A/A*	D	D
V153	Tanzanian	*T/T*	*A/A*	D	D
V155	Tanzanian	*T/T*	*A/A*	D	D
V156	Tanzanian	*T/T*	*A/A*	D	D
V158	Tanzanian	*T/T*	*A/A*	D	D
V160	Tanzanian	*T/T*	*A/A*	D	D
V163	Tanzanian	*T/T*	*A/A*	D	D
V166	Tanzanian	*T/T*	*A/A*	D	D
V167	Tanzanian	*T/T*	*A/A*	D	D
V169	Tanzanian	*T/T*	*A/A*	D	D
V171	Tanzanian	*T/T*	*A/A*	D	D
V172	Tanzanian	*T/T*	*A/A*	D	D
V174	Tanzanian	*T/T*	*A/A*	D	D
V175	Tanzanian	*T/T*	*A/A*	D	D
V176	Tanzanian	*T/T*	*A/A*	D	D
V177	Tanzanian	*T/T*	*A/A*	D	D
V180	Tanzanian	*T/T*	*A/A*	D	D
V182	Tanzanian	*T/T*	*A/A*	D	D
V185	Tanzanian	*T/T*	*A/A*	D	D
V186	Tanzanian	*T/T*	*A/A*	D	D
V189	Tanzanian	*T/T*	*A/A*	D	D

V192	Tanzanian	*T/T*	*A/A*	D	D
V178	Tanzanian	*T/T*	*A/A*	D	D
V194	Tanzanian	*T/T*	*A/A*	D	D
V195	Tanzanian	*T/T*	*A/A*	D	D
V196	Tanzanian	*T/T*	*A/A*	D	D
V197	Tanzanian	*T/T*	*A/A*	D	D
V198	Tanzanian	*T/T*	*A/A*	D	D
V199	Tanzanian	*T/T*	*A/A*	D	D
V201	Tanzanian	*T/T*	*A/A*	D	D
V202	Tanzanian	*T/T*	*A/A*	D	D
V203	Tanzanian	*T/T*	*A/A*	D	D
V205	Tanzanian	*T/T*	*A/A*	D	D
V206	Tanzanian	*T/T*	*A/A*	D	D
V207	Tanzanian	*T/T*	*A/A*	D	D
V208	Tanzanian	*T/T*	*A/A*	D	D
V209	Tanzanian	*T/T*	*A/A*	D	D
V211	Tanzanian	*T/T*	*A/A*	D	D
V213	Tanzanian	*T/T*	*A/A*	D	D
V215	Tanzanian	*T/T*	*A/A*	D	D
V216	Tanzanian	*T/T*	*A/A*	D	D
V217	Tanzanian	*T/T*	*A/A*	D	D
V218	Tanzanian	*T/T*	*A/A*	D	D
V219	Tanzanian	*T/T*	*A/A*	D	D
V220	Tanzanian	*T/T*	*A/A*	D	D
V221	Tanzanian	*T/T*	*A/A*	D	D
V222	Tanzanian	*T/T*	*A/A*	D	D
V223	Tanzanian	*T/T*	*A/A*	D	D
V224	Tanzanian	*T/T*	*A/A*	D	D
V225	Tanzanian	*T/T*	*A/A*	D	D
V226	Tanzanian	*T/T*	*A/A*	D	D
V227	Tanzanian	*T/T*	*A/A*	D	D
V228	Tanzanian	*TT*	*A/A*	D	D
V229	Tanzanian	*T/T*	*A/A*	D	D
V230	Tanzanian	*T/T*	*A/A*	D	D
V231	Tanzanian	*T/T*	*A/A*	D	D
V232	Tanzanian	*T/T*	*A/A*	D	D
V233	Tanzanian	*T/T*	*A/A*	D	D
V234	Tanzanian	*T/T*	*A/A*	D	D
V235	Tanzanian	*T/T*	*A/A*	D	D
V236	Tanzanian	*T/T*	*A/A*	D	D
V237	Tanzanian	*T/T*	*A/A*	D	D
V238	Tanzanian	*T/T*	*A/A*	D	D
V239	Tanzanian	*T/T*	*A/A*	D	D

V240	Tanzanian	*T/T*	*A/A*	D	D
V242	Tanzanian	*T/T*	*A/A*	D	D
V243	Tanzanian	*T/T*	*A/A*	D	D
V247	Tanzanian	*T/T*	*A/A*	D	D
V248	Tanzanian	*T/T*	*A/A*	D	D
V250	Tanzanian	*T/T*	*A/A*	D	D
V251	Tanzanian	*T/T*	*A/A*	D	D
V252	Tanzanian	*T/T*	*A/A*	D	D
V253	Tanzanian	*T/T*	*A/A*	D	D
V254	Tanzanian	*T/T*	*A/A*	D	D
V255	Tanzanian	*T/T*	*A/A*	D	D
V256	Tanzanian	*T/T*	*A/A*	D	D
V257	Tanzanian	*T/T*	*A/A*	D	D
V258	Tanzanian	*T/T*	*A/A*	D	D
V259	Tanzanian	*T/T*	*A/A*	D	D
V260	Tanzanian	*T/T*	*A/A*	D	D
V261	Tanzanian	*T/T*	*A/A*	D	D
V262	Tanzanian	*T/T*	*A/A*	D	D
V263	Tanzanian	*T/T*	*A/A*	D	D
V264	Tanzanian	*T/T*	*A/A*	D	D
V265	Tanzanian	*T/T*	*A/A*	D	D
V266	Tanzanian	*T/T*	*A/A*	D	D
V267	Tanzanian	*T/T*	*A/A*	D	D
V268	Tanzanian	*T/T*	*A/A*	D	D
V269	Tanzanian	*T/T*	*A/A*	D	D
V270	Tanzanian	*T/T*	*A/A*	D	D
V271	Tanzanian	*T/T*	*A/A*	D	D
V272	Tanzanian	*T/T*	*A/A*	D	D
V273	Tanzanian	*T/T*	*A/A*	D	D
V274	Tanzanian	*T/T*	*A/A*	D	D
V275	Tanzanian	*T/T*	*A/A*	D	D
V276	Tanzanian	*T/T*	*A/A*	D	D
V277	Tanzanian	*T/T*	*A/A*	D	D
V152	Tanzanian	*T/T*	*T/T*	P	P
V151	Tanzanian	*T/T*	*T/A*	T	T
V154	Tanzanian	*T/T*	*T/A*	T	T
V157	Tanzanian	*T/T*	*T/A*	T	T
V159	Tanzanian	*T/T*	*T/A*	T	T
V161	Tanzanian	*T/T*	*T/A*	T	T
V162	Tanzanian	*T/T*	*T/A*	T	T
V164	Tanzanian	*T/T*	*T/A*	T	T
V165	Tanzanian	*T/T*	*T/A*	T	T
V323	Tanzanian	*T/T*	*T/A*	T	T

V170	Tanzanian	*T/T*	*T/A*	T	T
V173	Tanzanian	*T/T*	*T/A*	T	T
V179	Tanzanian	*T/T*	*T/A*	T	T
V181	Tanzanian	*T/T*	*T/A*	T	T
V183	Tanzanian	*T/T*	*T/A*	T	T
V184	Tanzanian	*T/T*	*T/A*	T	T
V325	Tanzanian	*T/T*	*T/A*	T	T
V187	Tanzanian	*T/T*	*T/A*	T	T
V190	Tanzanian	*T/T*	*T/A*	T	T
V193	Tanzanian	*T/T*	*T/A*	T	T
V200	Tanzanian	*T/T*	*T/A*	T	T
V204	Tanzanian	*T/T*	*T/A*	T	T
V210	Tanzanian	*T/T*	*T/A*	T	T
V212	Tanzanian	*T/T*	*T/A*	T	T
V331	Tanzanian	*T/T*	*T/A*	T	T
V245	Tanzanian	*T/T*	*T/A*	T	T
V249	Tanzanian	*T/T*	*T/A*	T	T
V333	Tanzanian	*T/T*	*T/A*	T	T
V334	Tanzanian	*T/T*	*T/A*	T	T
V246	Tanzanian	*T/T*	*T/A*	T	T
V287	Tanzanian	*T/T*	*T/A*	T	T
V349	Tanzanian	*T/T*	*T/A*	T	T
V295	Tanzanian	*T/T*	*T/A*	T	T
V357	Tanzanian	*T/T*	*T/A*	T	T
CA14	URT Calabar	*C/T*	*T/A*	P	P

D – *dura*; T – *tenera*; P – *pisifera*. For (M1, M2), both *T/T* and *A/A* are $sh^{Deli\ Dura}/sh^{Deli\ Dura}$ as wild type; M1 - *T/C* and *C/C* are $sh^{Deli\ Dura}/sh^{MPOB}$ and sh^{MPOB}/sh^{MPOB}; M2 - *T/A* and *T/T* are $sh^{Deli\ Dura}/sh^{AVROS}$ and sh^{AVROS}/sh^{AVROS}.
